# Demonstration of the Coexistence of Duplicated LH Receptors in Teleosts, and Their Origin in Ancestral Actinopterygians

**DOI:** 10.1371/journal.pone.0135184

**Published:** 2015-08-13

**Authors:** Gersende Maugars, Sylvie Dufour

**Affiliations:** Muséum National d’Histoire Naturelle, Sorbonne Universités, Research Unit BOREA, Biology of Aquatic Organisms and Ecosystems, CNRS 7208-IRD 207-UPMC-UCBN, Paris, France; University of Rouen, France, FRANCE

## Abstract

Pituitary gonadotropins, FSH and LH, control gonad activity in vertebrates, via binding to their respective receptors, FSHR and LHR, members of GPCR superfamily. Until recently, it was accepted that gnathostomes possess a single FSHR and a single LHR, encoded by *fshr* and *lhcgr* genes. We reinvestigated this question, focusing on vertebrate species of key-phylogenetical positions. Genome analyses supported the presence of a single *fshr* and a single *lhcgr* in chondrichthyans, and in sarcopterygians including mammals, birds, amphibians and coelacanth. In contrast, we identified a single *fshr* but two *lhgcr* in basal teleosts, the eels. We further showed the coexistence of duplicated *lhgcr* in other actinopterygians, including a non-teleost, the gar, and other teleosts, e.g. Mexican tetra, platyfish, or tilapia. Phylogeny and synteny analyses supported the existence in actinopterygians of two *lhgcr* paralogs (*lhgcr1*/ *lhgcr2*), which do not result from the teleost-specific whole-genome duplication (3R), but likely from a local gene duplication that occurred early in the actinopterygian lineage. Due to gene losses, there was no impact of 3R on the number of gonadotropin receptors in extant teleosts. Additional gene losses during teleost radiation, led to a single *lhgcr* (*lhgcr1* or *lhgcr2*) in some species, e.g. medaka and zebrafish. Sequence comparison highlighted divergences in the extracellular and intracellular domains of the duplicated *lhgcr*, suggesting differential properties such as ligand binding and activation mechanisms. Comparison of tissue distribution in the European eel, revealed that *fshr* and both *lhgcr* transcripts are expressed in the ovary and testis, but are differentially expressed in non-gonadal tissues such as brain or eye. Differences in structure-activity relationships and tissue expression may have contributed as selective drives in the conservation of the duplicated *lhgcr*. This study revises the evolutionary scenario and nomenclature of gonadotropin receptors, and opens new research avenues on the roles of duplicated LHR in actinopterygians.

## Introduction

In teleost species, as in other vertebrates, reproduction is controlled by the brain-pituitary-gonad axis. The pituitary gonadotropins, follicle stimulating hormone (FSH) and luteinizing hormone (LH), are key hormones in the regulation of gametogenesis and steroidogenesis in both males and females [1]. Together with the pituitary thyrotropin (TSH), and the chorionic gonadotropin (CG) in primates, the gonadotropins are heterodimeric glycoproteins, composed of a common alpha subunit and a beta subunit that confers the biological specificity of the hormone [1–2]. After their characterization in mammals, LH and FSH have been demonstrated in the other tetrapods, as well as in teleosts and chondrichthyans, indicating the presence of the two gonadotropins in early gnathostomes [3].

LH and FSH exert their actions via binding to specific membrane receptors, LH receptor (LHR) and FSH receptor (FSHR), which belong to seven transmenbrane domain, G protein-coupled receptor (GPCR) superfamily, and to leucine-rich-repeat containing G protein-coupled receptor (LGR) subfamily [4]. CG acts via binding to the LH receptor, also named LHCGR [4]. Molecular studies by various research groups characterized a single *fshr* gene and a single *lhcgr* (synonymous *lhr*) gene in an increasing number of extant gnathostome species, including various teleosts [4–18], thus paralleling the duality of the pituitary gonadotropins [19].

Previous phylogeny studies of teleost gonadotropin receptor sequences, indicated that teleost LHR may separate into two clades [20–21]. Comparative genomics has revealed that a whole-genome duplication event (named 3R, for third round of genome duplication) occurred specifically in the teleost lineage [22]. The two LHR types may thus have resulted from the teleost 3R. As only one or the other of the two LHR types was found in given extant teleost species [21], this may suggest that the 3R would have been followed by alternative losses of one or the other *lhcgr* duplicated gene. Based on the finding that teleost dual *lhcgr* were mutually exclusive, and located on the same locus, another hypothesis was also proposed, that they may not result from gene duplication, but from interallelic conversion [21].

Our studies of the draft genomes of the European eel (*Anguilla anguilla*) and Japanese eel (*Anguilla japonica*), representative species of a basal group among teleosts (Elopomorphs) [23], revealed a larger conservation of duplicated genes in the eels, as compared to other teleost species. This is illustrated by a larger number of *hox* genes in the eels than in the other, more recently emerged, teleosts [24–25]. This also applies to genes involved in the neuro-endocrine systems. For instance, eels are so far the only teleost species to have conserved up to three kiss receptor genes [26]. Therefore in the present study, we investigated whether the eels may have conserved putative duplicated genes for gonadotropin receptors.

Previous studies in the Japanese eel, reported the partial cDNA cloning of a single *fshr* and a single *lhcgr* (*lhr*) [27], as well as the full-length cloning of cDNA encoding a single functional FSHR and a single functional LHR, respectively [28–30]. In the European eel, one full-length cDNA encoding a functional FSHR was also cloned and characterized [31]. Japanese eel *fshr* and *lhcgr* transcripts are expressed in female ovary and male testis and show expression changes during induced sexual maturation in both females and males [27,30].

In the present study, we successfully identified within the draft genomes of the European and Japanese eels, a single *fshr* gene, but two *lhcgr* genes, named *lhcgr1* and *lhcgr2*, providing the first evidence of the conservation of coexisting duplicated *lhcgr* in vertebrates. We isolated cDNA sequences of the single *fshr* and of the duplicated *lhcgr1* and *lhcgr2* in the European eel. Quantitative PCR (qPCR) revealed differential tissue expression patterns of *fshr* and of the two *lhcgr1* and *lhcgr2 mRNA*, which might have contributed to the maintenance of the duplicated *lhcgr* genes in the eels.

Based on our first evidence of two *lhcgr* in the eel, we reinvestigated the presence and number of gonadotropin receptor genes in other teleost species and found the conservation of two *lhcgr* in some other extant teleosts. These data open new research avenues for basic and applied fish reproductive endocrinology. To further elucidate the evolutionary history of gonadotropin receptors, we also identified new *fshr* and *lhcgr* sequences in genomes of key representative gnathostome species, including two chondrichthyans, the little skate (*Leucoraja erinacea*), and the elephant shark (*Callorhinchus milii*), a non-teleost actinopterygian which has diverged before the teleost-specific 3R, the spotted gar (*Lepisosteus oculatus*), and a representative species of an early diverging lineage among sarcopterygians, the coelacanth *(Latimeria chalumnae)*. Gene prediction, phylogeny and synteny analyses allowed us to propose a new scenario, suggesting that a single copy of *fshr* gene would have been conserved throughout vertebrate evolution, while duplicated *lhcgr* might have originated early in the actinopterygian lineage, before the teleost-specific third round of whole-genome duplication.

## Material and Methods

### Ethics statement

Complementary DNA cloning, and transcript tissue distribution studies in the European eel (*Anguilla anguilla*) were performed using total RNA samples, which had been already isolated and used in our previous studies [26,32–33], thus avoiding the sacrifice of additional eels, considered as endangered species.

### Gene and protein nomenclature

Gene and protein nomenclatures were standardized in the present text for all species by gene symbol in lowercase italic and protein symbol in uppercase non-italic.

### *In silico* prediction of gonadotropin receptor genes

#### Eel gonadotropin receptor genes

Genes encoding gonadotropin receptors were sought by Blast searches [34] using the Japanese eel (*Anguilla japonica*) *fshr* and *lhcgr* cDNA sequences (AB360713, EU635883, AY742794, AY742795, [27–29]), against the European and Japanese eel genomic sequence database (Eelgenome.org; NCBI) [24–25]. Exons and introns were annotated using the package CLC Main Workbench (CLC Bio, Qiagen, Denmark).

#### Gonadotropin receptors from other teleosts

Using the FSHR and the two LHR protein sequences identified in the European eel in this study as queries (tBlastn), *fshr* and *lhcgr* genes were sought and annotated (if not yet predicted) in the increasing number of teleost genomes available in Ensembl or NCBI (see S1 Table).

#### Gonadotropin receptor genes from other vertebrates

In addition to the already known vertebrate gonadotropin receptors, *fshr* and *lhcgr* genes were also sought in genomes of other vertebrate species of key-phylogenetic positions (S1 Table): in two chondrichthyans, an elasmobranch, the little skate, *Leucoraja erinacea* and a holocephalan, the elephant shark, *Callorhinchus milii*, in a basal sarcopterygian fish, the coelacanth, *Latimeria chalumnae*, in various sauropsids including avians, crocodilians, chelonians and squamates (e.g. chicken, *Gallus gallus*; white-throat sparrow, *Zonotrichia albicollis*; American alligator, *Alligator mississipiensis*; Chinese alligator, *Alligator sinensis*; Chinese soft-shelled turtle, *Pelodiscus sinensis;* painted turtle, *Chrysemys picta*; green anole, *Anolis carolinensis*; Burmese python, *Python bivittatus*; king cobra, *Ophiophagus hannah*) and in a non-teleost actinopterygian, the spotted gar, *Lepisosteus oculatus*.

Gene coding sequences (CDS) were annotated or corrected by comparison with well-known orthologous CDS from other species, in respect with the canonical splice site rule using CLC Main Workbench (S1 Table).

### cDNA cloning of European eel gonadotropin receptors

The predicted European eel *fshr*, *lhcgr1* and *lhcgr2* CDS sequences were completed and confirmed by cDNA cloning. Specific cloning primers were designed on both extremities and inside the predicted CDS sequences, using Primer3 browser [35] (S2 Table). First strand cDNAs were synthesized from total RNA samples previously prepared from testis and ovary of silver European eels [26,33]. One μg was reverse transcribed using superscript III (Invitrogen, Carlsbad, CA, USA), random primers (50 ng) (Invitrogen) and oligo(dT)_18_ (50 μg) (Roche, Mannhein, Germany), at 50°C for 60 min, after an initial step at 25°C for 10 min. PCR were performed using GoTaq mix (Promega, Fitchburg, WI, USA) on ovary and testis cDNAs. PCR amplifications were performed on the Life Express Thermal Cycler (Hangzhou Bioer Technology Co., Ltd, Hangzhou, China) with the following thermal conditions: after an initial step of 95°C for 10 min, five cycles at 95°C for 30 sec, annealing temperature ranging from 65°C to 62°C for 30 sec, 72°C for 2 min, followed by 30–40 cycles with annealing temperature ranging from 55 to 53°C for 30 sec, and a final extension at 72°C for 5 min. *Lhcgr1* cDNA was cloned from the ovary, *fshr* and *lhcgr2* cDNAs from the testis. For *lhcgr2* cloning, cDNA templates were previously denaturated during 10 min at 95°C. Amplified products were separated on agarose gel and purified using QIAquick gel extraction kit (Qiagen, Vento, Netherlands). Purified PCR fragments were sequenced directly, or after subcloning into a pGEM-T Easy Vector (Promega), by the sequencing service of GATC Biotech (Constance, Germany).

### Prediction of gonadotropin receptor protein sequences and domains

Nucleotide sequence translation and first methionine were predicted using the package CLC Main Workbench. The signal peptide cleavage site was determined using SignalP [36] and putative transmembrane helices were predicted using TOPCONS [37]. Searches for motifs and sequence patterns were performed by comparison with protein families databases on ExPASy server [38]. Leucine-rich repeats were identified using Pfam database [39] and the recent crystallography study of human FSHR extracellular domain [40–41]. Potential tyrosine sulfation sites were predicted by sulfosite browser with 80% prediction sensitivity [42] and palmitoylation sites in the ICD were predicted using PalmPred browser [43].

### Phylogeny analysis and domain comparison of gonadotropin receptors

Seventy eight gonadotropin receptor sequences of representative vertebrates were collected from the available databases of NCBI and Ensembl, including 33 sequences defined in the present study and 45 sequences previously published (S1 Table). Multiple alignments were performed using ClutstalW and were manually adjusted taking into account the receptor topology to improve the alignment accuracy. The phylogeny of gonadotropin receptors was inferred by Maximum Likelihood using PhyML with the WAG model of substitution [44] combined to the neighbor-joining interchange (NNI) method on Seaview browser [45]. A tree was generated and robustness of the phylogeny assumption was evaluated by the approximate likelihood test (aLRT) SH-like branch support and by bootstrapping procedure from 500 data set replicates. The sequence of the glycoprotein hormone receptor of an early chordate, the lancelet (*Branchiostoma floridae*), *(XP_002610242)* was designated to root the tree (S1 Table).

### Synteny analysis of gonadotropin receptors

Flanking genes of the three gonadotropin receptors in European and Japanese eel draft genomes were sought by Blast searches. Only a limited number of flanking genes could be annotated due to the current size of the scaffolds. The Spotted gar, a non-teleost actinopterygian, which we showed to possess *fshr*, *lhcgr1 and lhcgr2*, was chosen as reference to study the genomic regions of the gonadotropin receptors. At the beginning of our study, the spotted gar genome was assembled at the level of chromosome but was non-annotated. The flanking genes of the spotted gar gonadotropin receptors were identified by blasting the contigs (AHAT01001660 to AHAT01001713) against the zebrafish protein dataset, using CLC Main Workbench. In the course of our study, an annotated version of the spotted gar genome became available in Ensembl and NCBI and was in agreement with our gene synteny predictions.

In addition to the spotted gar, *fshr*, *lhcgr1 and lhcgr2* syntenic regions were compared using Genomicus (version 7.5) [46], Ensembl and NCBI genome visualization browser, in teleost species that have retained either one or both duplicated *lhcgr1* and *lhcgr2*, in representative tetrapods, including human, and in a basal sarcopterygian, the coelacanth.

### Tissue distribution of gonadotropin receptors transcript levels in the European eel

#### RNA samples and cDNA synthesis

Tissue distribution analysis was performed on RNA samples from 8 female silver migrating eels caught in the Loire River in France and previously prepared [32–33]. Total RNA extracted from ovary, olfactory bulb, mesencephalon and diencephalon, telencephalon, cerebellum, medulla oblongata, pituitary, eyes, liver, intestine, muscle, adipose tissue, gills, and thyroid follicles were used. Previously prepared RNA samples from testis [26] from 8 male silver eels were also analyzed. The silver stage is the last continental phase of the eel life cycle. The silver eels are ready to perform the oceanic reproductive migration, but are still immature, the ovary being at the pre-vitellogenic stage, and the testis at the spermatogonial stage. Four hundred ng of total RNA were reverse transcribed using Superscript III (Invitrogen) and random primers (50 ng) (Invitrogen), at 50°C for 60 min after an initial step of 25°C for 10 min.

#### Quantitative PCR primer design and analyses

Eel specific primer sets for each gonadotropin receptor were designed using Primer3 [35] on two successive exons, or on exon junctions, of the extracellular domain. Various primer set combinations were tested and compared. Efficiency and amplification specificity were checked for each primer set. The sets with the higher efficiency (100% for *fshr* and *lhcgr1* and 87% for *lhcgr2*) were chosen for the following quantification experiment (S2 Table). Quantitative PCR (qPCR) product was sequenced to control the primer specificity.

Messenger RNAs were assayed using Light Cycler System (Roche) with the LightCycler FastStart Master plus SYBR Green I kit (Roche) as recommended by the manufacturer. The final primer concentration used was 500 nM. Each sample was run in duplicate. The PCR conditions were 95°C for 10 min followed by 50 cycles at 95°C for 5 sec, 60°C for 10 sec and 72°C for 10 sec. The specificity of amplified qPCR products, was checked by melting curve analysis. Relative transcript levels were quantified using standard curves prepared with cDNA from tissue samples in which they were abundant, *i*.*e*. ovarian, telencephalon, or testis, for *lhcgr1*, *lhcgr2*, and *fshr*, respectively. Results were expressed as arbitrary units of gonadotropin receptor transcript level / total RNA level, and normalized to the mean expression value in the ovary, considered as 1.

## Results, Discussion, and Conclusions

### A single *fshr* but two *lhcgr* are present in basal teleosts, the eels

#### Prediction of a single fshr gene in both European and Japanese eel draft genomes

Blast searches using the *fshr* cDNA sequence cloned in the Japanese eel (AB360713; [28]) allowed us to retrieve one scaffold covering the full-length *fshr* cDNA in the Japanese eel genome, as well as in the European eel genome (S1 Fig).

#### Prediction of two lhcgr genes in both European and Japanese eel draft genomes

Blast of the *lhcgr* cDNA sequences previously reported in Japanese eels (AY742795; [27] Jeng et al. 2007 and EU635883; [29]) against the Japanese eel genome, allowed the prediction of two *lhcgr* genes located on two distinct scaffolds (S1 Table). One *lhcgr* gene corresponds to the cDNA sequence identified by Kazeto *et al*. [29]. We named it *lhcgr1 a*ccording to phylogenic and synteny analyses (see sections below). The other *lhcgr* showed high sequence identity with the partial cDNA sequence identified by Jeng et al. [27] and was named *lhcgr2*. Blast of the two Japanese eel predicted *lhcgr* sequences against the European eel genome also allowed the identification of two *lhcgr* genes located on two distinct scaffolds. European eel *lhcgr2* sequence could be only partially predicted, due to low assembly resolution or ambiguities of the European eel draft genome. To confirm the presence of three gonadotropin receptors in the eels and complete the predicted coding sequences, the cDNA for *fshr*, *lhcgr1* and *lhcgr2* were cloned in the European eel.

#### Cloning of the cDNAs of the three European eel gonadotropin receptors

*Fshr* cDNA (2040 bp) was cloned using testis RNA (S2 Fig). The cloned sequence shared 99.7% identity with the predicted European eel *fshr* CDS and the recently published European eel *fshr* cDNA cloned from ovarian RNA by Minegishi et al. [31] (AB700600). Few mismatches observed between the predicted genes and the corresponding transcripts may reflect some polymorphism. These differencies concerned the third base position of the codons, with no impact on amino acid sequences. A partial cDNA of 1913 bp encoding *lhcgr1* was cloned from ovarian RNA (S3 Fig). This sequence covered 90.4% of the predicted CDS (2115 bp) of European eel *lhcgr1*. It shared 99% identity with the European eel *lhcgr1* CDS and 98.1% identity with the Japanese eel *lhcgr1* cDNA (EU635883; [29]). The full-length coding sequence of the European eel *lhcgr2* was cloned (2104 bp) using testis RNA (S4 Fig). It shared 98.5% identity with our predicted Japanese eel *lhcgr2* CDS. It is the first time that duplicated *lhcgr* transcripts are isolated in a vertebrate species.

#### Prediction of the protein domains of the eel gonadotropin receptors

**Fig 1:**
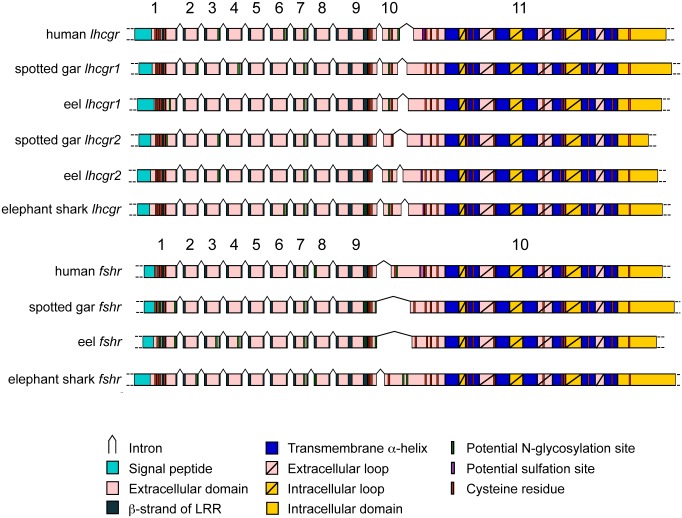
Gene organization of gonadotropin receptors in human, eel, spotted gar and elephant shark. Numbers cover the 11 exons of the *lhcgr* and the 10 exons of the *fshr*. On the coding sequences are indicated: signal peptide, extracellular domain including β-strand of leucin-rich repeat (LRR), transmembrane helices (TH), intracellular and extracellular loops (il and el, respectively) and intracellular domain. Cysteine residues, potential N-glycosylation and sulfation sites are also illustrated.

**Fig 2:**
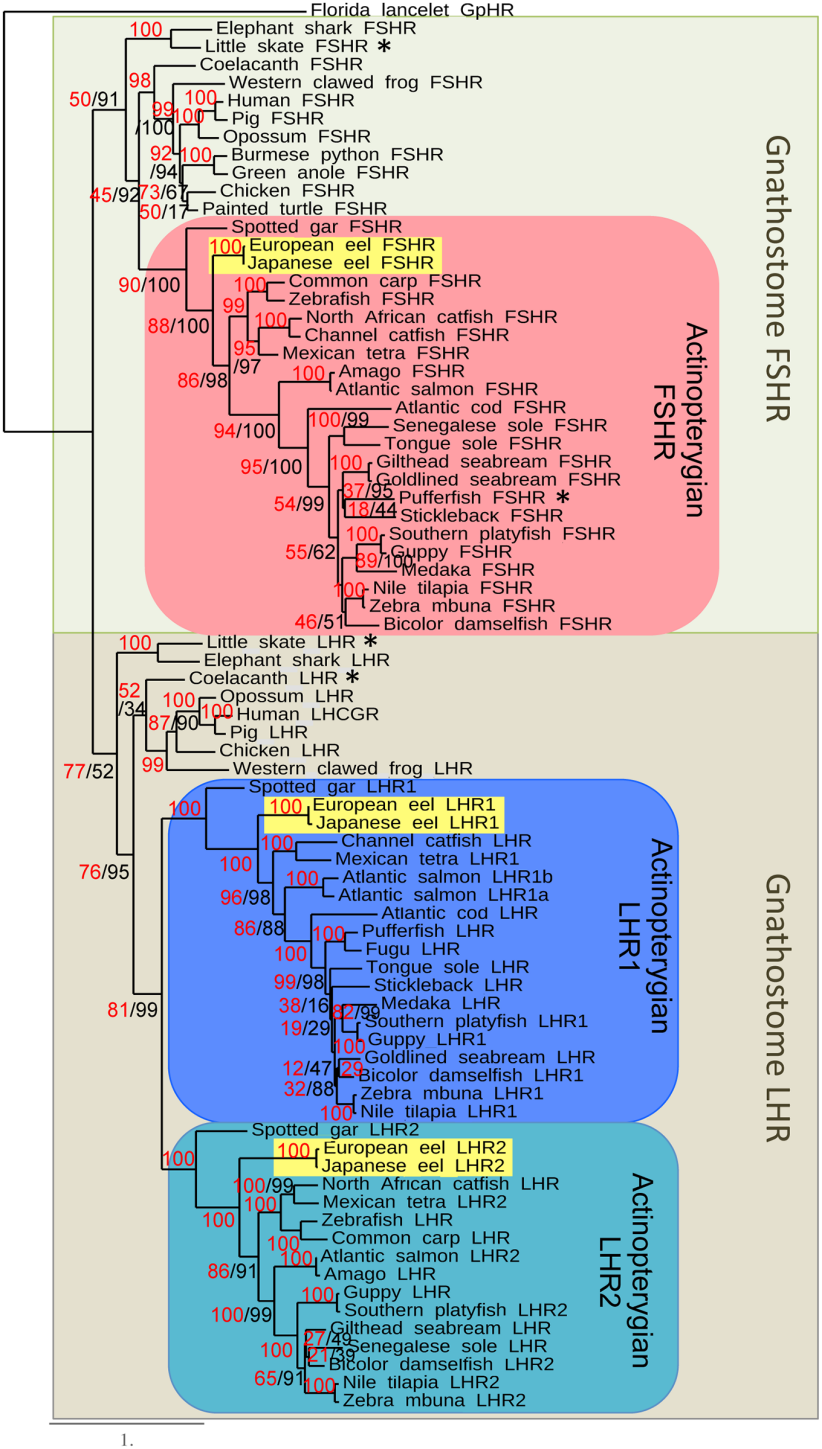
Phylogeny relationships of the gonadotropin receptors. Phylogram of maximum likelihood relationships between LHR and FSHR amino acid sequences of representative gnathostome species. Bootstrap values over 500 replicates (%) are given next to each node in red and SH-like aLRT values (%) are given in black (when different from the boostrap value). Asterisks indicate partial sequences. The actinopterigyan FSHR and LHR clades are highlighted to facilitate the phylogram examination. The sequence of the glycoprotein hormone receptor (GpHR) of an early chordate, the lancelet, was designated to root the tree.

European eel *fshr* CDS encoded a 660 amino acids (aa) FSHR including a 17 aa signal peptide (S2 Fig). *Lhcgr1* and *lhcgr 2* CDS encoded a 704 aa LHR1 including a 26 aa signal peptide, and a 700 aa LHR2 including a 17 aa signal peptide, respectively (S3 and S4 Figs). The three eel gonadotropin receptors showed the typical topology of the glycoprotein hormone receptors with a long N-terminal extracellular domain (ECD), corresponding to about the half of the whole amino acid sequence (322 aa, 349 aa and 359 aa, for FSHR, LHR1 and LHR2, respectively) (Fig 1). The N-terminal cysteine box (cb1) of ECD of the eel receptors included 4 cysteines for both eel LHR as for mammalian glycoprotein hormone receptors, but only 2 cysteines for FSHR as in some other teleosts. The typical leucine-rich repeat domain (LRRD) of the ECD was composed of 11 leucine-rich repeats (LRR) for the three eel receptors as in mammals (Figs 1 and 2). The succession of LRR confers to the ECD a concave shape that is involved in the hormone binding [40–41,47–50]. The C-terminal part of the ECD, which corresponds to the hinge region, was delimited in the three eel receptors by two conserved cysteine boxes (cb2 and cb3) containing 3 cysteines each. The transmembrane domain (TMD) of the three eel receptors (263 aa, 267 aa, and 264 aa, for FSHR, LHR1 and LHR2 respectively) was composed, as for all GPCR, of highly conserved seven transmembrane alpha-helices (TH), connected by 3 extracellular (el) and 3 intracellular (il) loops. The receptors terminated with an intracellular domain (ICD) corresponding to a C-terminal cytosolic tail (58 aa, 62 aa, and 60 aa, for FSHR, LHR1 and LHR2 respectively). The ICD is involved in GPCR signal transduction and receptor intracellular trafficking and desensitization (for review [51]).

#### Comparison of the gene structure of the three eel gonadotropin receptors

Complementary DNA sequences allowed us to assess the intron-exon gene structure of the eel gonadotropin receptors. The European eel *fshr* gene consisted of 10 exons and showed the typical exon-intron structure described for the human *fshr* [52], (Fig 1). The coding part of first exon (156 bp) encoded the signal peptide and the N-terminal region of the ECD, including the first cysteine box (cb1) and one of the LRR. A succession of 7 small exons (69-81bp) and a larger 9th exon (188 bp) encoded the major part of the ECD, including all the other LRRs, and two cysteines of the second cysteine box (cb2). The large 3’-terminal “rhodopsin like” exon, covered the C-terminal region of the ECD, including the third cysteine of the cb2, the hinge region and the cb3, together with the TMD and ICD. Prediction of the *fshr* gene structure in other vertebrates such as chondrichthyans, non-teleost actinopterygian and various teleosts (Fig 1 and S1 Table) showed a similar typical 10 exon structure, with the exception of salmonid and percomorph *fshr*, which present several additional exons as previously reported [21,53].

The European eel duplicated *lhcgr* genes were both organized in 11 exons as the human *lhcgr* (Fig 1) [4,54]. As compared to eel *fshr* gene, they included an additional exon between the 9th exon and the large terminal exon. As for human *lhcgr*, this additional small exon (72 bp and 69 bp for eel *lhcgr1* and *lhcgr2*, respectively) encoded the third cysteine of the cb2, and a part of the hinge region. This typical 11-exon structure was also predicted for *lhcgr* from various vertebrates, including chondrichthyans, a non-teleost actinopterygian and various teleosts [21] (Fig 1 and S1 Table) (see next section).

### Identification of additional gonadotropin receptors in representative gnathostome genomes

To investigate the evolution of the gonadotropin receptors in gnathostomes, we conducted a search for gonadotropin receptor sequences within 30 vertebrate genomes including two chondrichthyans, one basal sarcopterygian fish, 12 tetrapods, 1 non-teleost actinopterygian and 14 teleosts.

#### A single fshr and one or two lhcgr are present in teleosts

The screening of the available representative teleost genomes revealed, as in eels, the presence of one *fshr* and of both *lhcgr1* and *lhcgr2* genes in seven other species, *e*.*g*. in a characiforme, the Mexican tetra, *Astyanax mexicanus*, in a salmonid, the Atantic salmon, *Salmo salar*, in two atherinomorphs, the platyfish, *Xiphophorus maculatus* and the guppy, *Poecilia reticula*, and two cichlids, the Nile tilapia, *Oreochromis niloticus*, and zebra mbuna, *Maylandia zebra*, and in a pomacentridae, the bicolor damselfish, *Stegastes partitus* (S1 Table). The coexistence of duplicated *lhcgr* in various teleost species clearly refutes one previous assumption of two mutually exclusive *lhcgr* in this lineage [21]. In Atlantic salmon, in addition to the *lhcgr2* gene, we succeeded to annotate duplicated *lhcgr1* genes (S1 Table and S5 Fig). The large number of *lhcgr1* in this species may result from the recent salmonid tetraploidization (4R) [55].

In contrast, we found a single *fshr* gene and alternatively only one or the other of the two *lhcgr* genes, in seven other teleost annotated genomes. One *lhcgr* homologous to eel *lhcgr2* (see section Phylogeny) was identified in a cypriniforme, the zebrafish, *Danio rerio*, while *lhcgr* homologous to eel *lhcgr1* (see section Phylogeny) was identified in a gadiforme, the Atlantic cod, *Gadus morua*, in a beloniformes, the Japanese medaka, *Oryzias latipes*, in a gasterosteiforme, the stickleback *Gasterosteus aculeatus*, and in two tetraodontiformes, the pufferfish, *Tetraodon nigroviridis* and the fugu, *Takifugu rubripes*. In another cypriniforme, the common carp, *Cyprinus carpio carpio*, two putative *lhcgr2* were identified (one full-length and one partial with potential frameshifts), that may result from the recent cyprinid tetraploidization (4R) [56–57] (S1 Table).

#### A single fshr and two lhcgr are present in a non-teleost actinopterygian

We could predict one *fshr* and two *lhcgr* (S1 Table), homologous to eel *lhcgr1* and *lhcgr2*, respectively (see section Phylogeny), in the genome of a holostean actinopterygian, the spotted gar. The *fshr* consisted of a succession of 10 exons, as human and eel *fshr* (Fig 1), and encoded a 719 aa receptor. Both *lhcgr* were organized in 11 exons as eel *lhcgr1* and *lhcgr2*, and human *lhcgr* (Fig 1), and encoded a LHR1 (696 aa) showing 53% identity with eel LHR1, and a LHR2 showing 59% identity with eel LHR2, respectively (Table 1). Considering that the holostean lineage has diverged before the teleost-specific 3R, the duality of *lhcgr* in the gar indicates that a gene duplication event has already occurred before the teleost 3R.

**Table 1:** Overall and subdomain amino acid sequence comparison between the European eel LHR1, LHR2 and FSHR and representative species (% of identity).

	*Overall*			*ECD*			*TMD*			*ICD*		
	LHR1	LHR2	FSHR	LHR1	LHR2	FSHR	LHR1	LHR2	FSHR	LHR1	LHR2	FSHR
**European eel LHR1**		49.7	44.9		43.6	34.1		68.4	69.9		16.9	13.5
**European eel LHR2**	49.7		42.2	43.6		32.1	68.4		63.9	16.9		17.1
**European eel FSHR**	44.9	42.2		34.1	32.1		69.9	63.9		13.5	17.1	
***Actinopterygian* LHR1**												
Spotted gar	59.6	47.9	47.6	56.5	44.0	34.4	73.3	63.5	74.8	27.2	18.9	25.6
Japanese eel	**98.2**	49.5	44.7	**98.4**	43.1	33.9	**98.5**	68.8	70.2	**95.5**	15.6	10.8
Mexican tetra	64.2	47.5	43.5	57.8	44.0	31.9	78.2	61.2	67.1	46.5	19.2	20.8
Japanese medaka	59.4	44.8	41.2	49.7	40.0	30.1	77.5	60.1	65.7	44.4	18.0	13.0
Southern platyfish	60.8	46.7	41.9	51.5	42.2	32.2	79.0	60.8	64.1	43.6	21.8	14.3
Ntile ilapia	61.7	46.0	42.6	52.3	41.7	32.7	77.1	59.7	65.7	49.3	21.8	13.0
***Actinopterygian* LHR2**												
Spotted gar	48.6	54.0	46.6	43.2	50.8	34.5	64.9	65.4	71.0	22.2	28.6	23.7
Japanese eel	49.6	**97.9**	42.0	43.6	**97.4**	31.5	68.1	**98.9**	63.9	16.9	**96.7**	18.6
Zebrafish	47.2	57.6	43.6	40.3	52.0	32.2	65.7	73.0	68.7	19.5	32.0	14.3
Mexican tetra	47.9	56.9	43.0	42.9	52.3	32.2	66.8	72.6	67.9	14.6	26.6	15.8
Southern platyfish	45.5	54.4	42.0	39.4	46.5	30.3	65.7	73.4	66.8	11.9	28.9	13.6
Nile tilapia	41.5	54.5	42.3	41.0	49.4	30.8	65.3	72.6	66.8	15.6	14.7	15.4
***Sarcopterygian* LHR**												
Human	47.6	46.4	46.9	40.0	41.9	34.1	66.8	61.6	71.0	21.7	18.8	28.8
Chicken	48.4	45.5	46.4	40.7	40.3	33.2	68.7	62.7	72.9	22.6	16.9	27.1
Xenopus	45.4	44.6	45.6	36.0	37.8	32.3	68.3	63.5	72.4	18.8	17.1	25.6
Coelacanth	47.5*	43.9*	46.2*	38.2*	35.7*	32.0*	69.9	63.5	73.3	22.4	21.4	26.6
***Chondrichthyan* LHR**												
Elephant shark	45.6	46.0	47.1	38.7	39.8	34.7	65.7	64.3	73.3	14.8	17.5	21.1
***Actinopterygian* FSHR**												
Spotted gar	43.2	40.4	66.1	33.9	31.3	61.6	69.1	62.7	77.9	11.1	14.6	48.2
Japanese eel	44.8	42.0	**99.2**	33.6	31.6	**99.1**	70.2	64.3	**99.6**	13.5	17.1	**98.3**
Zebrafish	43.6	43.1	70.1	31.5	32.8	59.3	69.1	64.6	82.8	16.2	20.0	77.6
Mexican tetra	44.6	43.0	70.5	34.1	32.8	62.5	68.7	64.6	81.7	15.6	18.3	68.9
Japanese medaka	40.5	38.4	53.4	28.1	27.5	39.7	67.6	61.2	75.2	14.3	18.7	45.5
Southern platyfish	40.2	38.7	56.0	28.1	27.5	43.8	67.6	62.7	76.3	11.7	17.3	45.5
Nile tilapia	41.5	38.3	55.8	39.1	26.3	42.5	68.7	62.7	77.5	15.6	20.0	47.0
***Sarcopterygian* FSHR**												
Human	43.2	41.6	54.7	32.6	32.2	42.0	66.8	62.7	75.1	19.0	18.1	47.0
Chicken	42.3	40.3	55.7	30.6	29.5	42.0	67.6	62.4	75.6	18.2	21.1	56.3
Xenopus	43.6	40.2	54.6	31.3	30.6	40.1	69.9	61.2	76.7	18.7	18.6	51.6
Coelacanth	44.6	43.1	56.9	33.8	33.2	43.0	69.1	64.6	76.7	17.3	21.1	58.1
***Chondrichthyan* FSHR**												
Elephant shark	43.3	41.4	49.5	33.6	32.9	36.4	68.7	63.5	75.2	14.7	15.6	29.2

Asterisk (*) indicates partial sequence. Comparisons of FSHR, LHR1 and LHR2 amino acid full-length and subdomains sequences between European eel and Japanese eel are indicated in bold.

#### A single fshr and a single lhcgr are present in sarcopterygians

In a basal sarcopterygian, the coelacanth, one partial *lhcgr* could be predicted on scaffold JH127072 (S1 Table). This partial sequence consisted of 10 exons giving a 632 aa protein, the first exon encoding for the signal peptide being missing. In addition, we could identify one *fshr* gene (S1 Table) on scaffold JH127072, positioned in tandem with the *lhcgr*. The *fshr* sequence consisted of 10 exons and encoded for a 690 aa receptor. As in the coelacanth, a single *fshr* and a single *lhcgr* are present in the other sarcopterygians, including tetrapods. No additional *lhcgr* could be found in any sarcopterygians. In the course of our investigation of genome databases, we observed that some sauropsid species possess *fshr* but may have lost *lhcgr*. In birds, *fshr* and *lhcgr* are still present and positioned in tandem, as in coelacanth and mammals. In contrast, we could not find any *lhcgr* in the genome of three squamates, the anole lizard, the Burmese python and the king cobra. Furthermore, we found several premature stop codons and frameshifts in the putative *lhcgr* sequences of a chelonian, the painted turtle and of crocodilians, the Chinese and American alligators (S1 Table). This suggests that a loss of a functional LHR would have occurred several independent times during the sauropsid radiation, in squamate, chelonian and crocodilian lineages [58–59]. Similarly, our recent studies on the kisspeptin system revealed multiple and independent losses of *kiss* and *kiss receptor* genes during the sauropsid radiation [60].

#### A single fshr and a single lhcgr are present in chondrichthyans

In the elephant shark genome, a single *fshr* and a single *lhcgr* could be predicted on scaffold 134 and scaffold 49, respectively (S1 Table). They showed the typical gene organization consisting of 10 exons and 11 exons (Fig 1), respectively. *Fshr* encoded a 741 aa receptor, and *lhcgr* a 709 aa receptor. From the little skate contig data, we succeeded to assemble a single partial *fshr* encoding 570 aa and a single *lhcgr* encoding 691 aa (S1 Table).

In conclusion, genome searches indicated that among all gnathostomes investigated, the actinopterygians, including teleost and non-teleost species, were the only group with two *lhcgr*. This suggests that duplicated *lhcgr* may represent a specific feature of the actinopterygian lineage.

### Phylogeny of gonadotropin receptors

To better understand the relationships between gnasthostome gonadotropin receptors, a molecular phylogeny (Fig 2) analysis was conducted on 78 gonadotropin receptor protein sequences from 35 species (S1 Table). The glycoprotein hormone receptor of a cephalochordate, the lancelet, was used to root the tree. The gnathostome FSHR and LHR formed two distinct monophyletic groups (Fig 2).

The FSHR from the two chondrichthyan species clustered together at the base of the gnathostome FSHR group. The osteichthyan FSHR subdivided in two clades, one formed by the sarcopterygian and the other by the actinopterygian FSHR. Among the sarcopterygians, the coelacanth FSHR branched at the basis of the tetrapod FSHR, in respect to its phylogenic position. Similarly, among the actinopterygians, the spotted gar FSHR branched at the basis of teleost FSHR, in agreement with its phylogenic position. The European and Japanese eel FSHR branched at the basis of the other teleost FSHR, consistent with their corresponding phylogenic position. The topology of the teleost FSHR clade followed the known phylogenetic relationships among teleosts.

The LHR from the two chondrichthyan species formed a distinct clade at the base of all gnathostome LHR, in respect to their phylogenic position. The osteichthyan LHR group subdivided in two clades: one clade of sarcopterygian LHR including the coelacanth LHR and the tetrapod LHR and one clade of actinopterygian LHR. The actinopterygian LHR clearly further separated into two clades, each well supported (99–100% bootstrap values), and including both gar and eel LHR1, or gar and eel LHR2. Spotted gar, European and Japanese eel LHR1 or LHR2 were at the basis of each of the two actinopterygian LHR clades, respectively, in agreement with their phylogenic positions. For other teleost species possessing two LHR, such as Atlantic salmon, Mexican tetra, Nile tilapia and platyfish, the duplicated LHR were distributed between actinopterygian LHR1 and LHR2 clades, respectively, as for eels and spotted gar. In the species where only a single LHR was found in the genome, such as zebrafish, carp, medaka, stickelback, tetraodon, fugu and cod, the LHR clustered in one or the other of the two clades (LHR2 for cyprinids, LHR1 for the other species). Previously characterized LHR from various teleost species (*e*.*g*., siluridae, pleuronectidae, sparidae), for which the genome has not yet been completed, were represented in one or the other LHR clade, with a topology congruent with the known phylogeny of the teleost radiation. One may highlight the striking case of some closely related species, such as siluriformes [20], with the LHR of the channel catfish, *Ictalurus punctatus*, branching within the LHR1 clade, while the LHR of the African catfish, *Clarias gariepinus*, within the LHR2 clade. A similar situation was previously reported in percomorphs, between two pleuronectiforms or between two sparidae [21]. Our finding of coexisting duplicated *lhcgr* should promote further investigation to decipher if each of these species possess in fact the two *lhcgr* receptors, or if alternative losses of one or the other *lhcgr* have occurred in closely related species.

In conclusion, the presence of duplicated *lhcgr* in the eel provides the first demonstration of the duality of *lhcgr* in teleosts, as previously suggested by Chauvigné et al. [21]. Furthermore, we showed the presence of duplicated *lhcgr* in various other teleosts, including characids, salmonids, cichlids, poecilids. The presence of duplicated *lhcgr* genes in teleosts may have led to the assumption that they are derived from the teleost-specific third whole-genome duplication (3R). We recently demonstrated a 3R-origin for other duplicated glycoprotein hormone receptors in teleosts, thyrotropin hormone receptors (*tshra* and *tshrb*) [33]. However, differently from the case of *tshr*, our present study revealed that the duplicate *lhcgr1* and *lhcgr2* genes are also present in a non-teleost actinopterygian, the spotted gar, suggesting that the *lhcgr* duality in teleosts does not result from the 3R but might be derived from an anterior duplication event. Given the presence of a single *lhcgr* gene in chondrichthyans and sarcopterygians including the basal coelacanth, the most parsimonious assumption is that the *lhcgr* duplication event is specific to the actinopterygian lineage. In teleosts, the 3R whole-genome duplication event would have been expected to generate further duplicated *fshr*, *lhcgr1* and *lhcgr2* genes. Given the fact that, despite the 3R, we found no additional *fshr* and *lhcgr* genes in extant teleosts as compared to the gar, it could be assumed that a massive loss of duplicated gonadotropin receptor genes derived from the 3R, occurred in early teleosts. The impact of the recent tetraploidization events (4R) that occurred in salmonids, is still evident on the number of *lhcgr* genes in Atlantic salmon (Fig 2).

### Synteny of gonadotropin receptors

**Fig 3:**
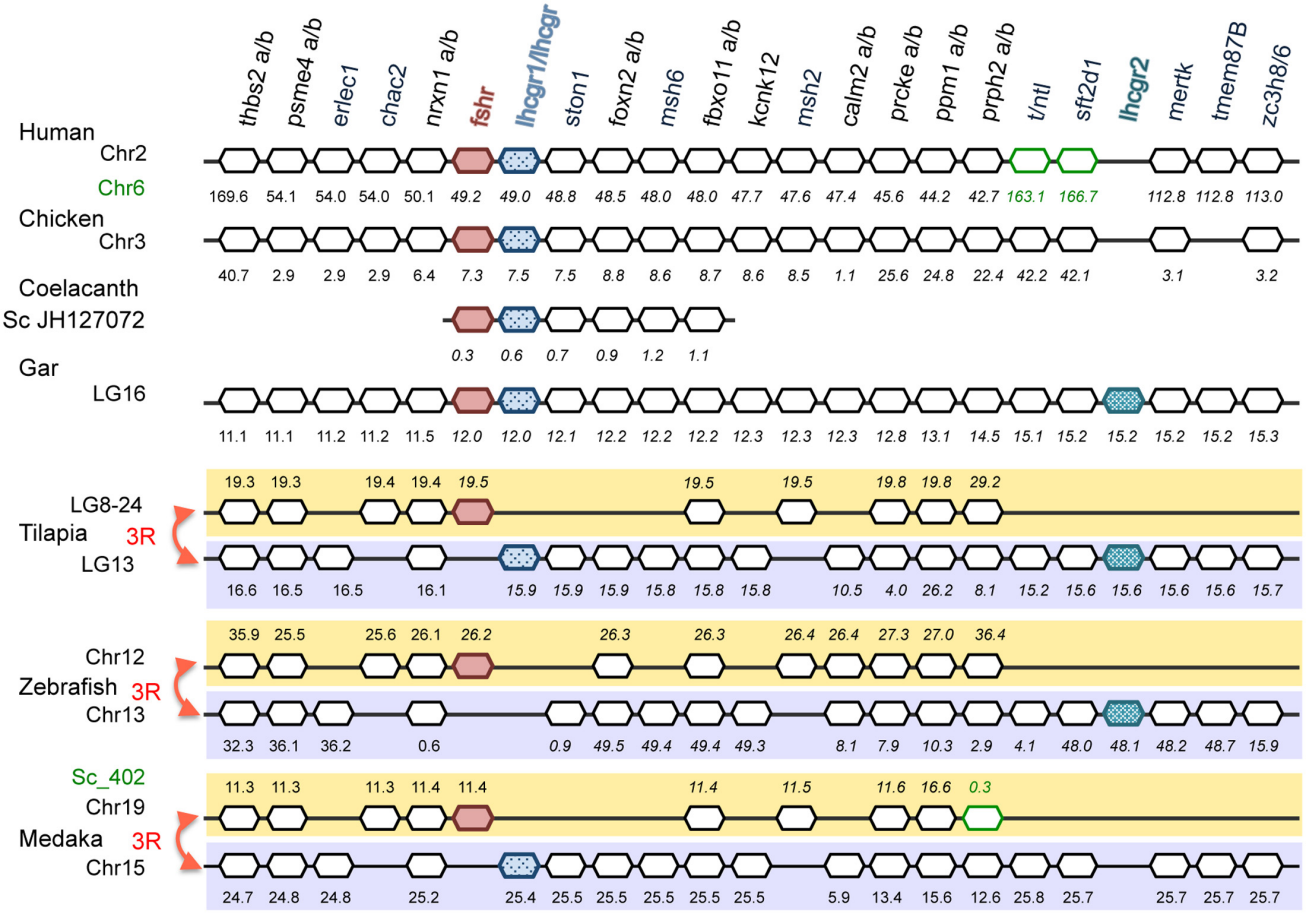
Syntenic analysis of gonadotropin receptor genomic region. Genomic regions flanking *fshr* and *lhcgr* genes were analysed in representative vertebrate species including sarcopterygians (human, chicken, coelacanth) and actinopterygians (spotted gar, tilapia, zebrafish, medaka) by using the region overview on the Ensembl or NCBI genome browsers. The chromosome number is indicated beside the species name. *Fshr* genes are indicated in red, *lhcgr/lhcgr1* in light blue and *lhcgr2* in darker blue. The symbols of the genes of interest present on another genomic region in some species are indicated in green. Genes are named according to the Ensembl nomenclature. Gene positions are given in Mega base below the symbol of the genes.

To further elucidate the gonadotropin receptor evolutionary history, we characterized and compared the adjacent genomic regions of each *fshr* and *lhcgr* loci, in sarcopterygians including representative tetrapods and the basal coelacanth, in a non-teleost actinopterygian, the spotted gar, and in teleosts including species like the eels and the tilapias which have conserved both *lhcgr* types, and species like the medaka and the zebrafish that have conserved only a single *lhcgr* (*lhcgr1* or *lhcgr2*) (Fig 3).

In human genome, which contains only a single *fshr* and a single *lhcgr* as for all sarcopterygians, both receptors are positioned in tandem within the same locus (locus *2p21*) [61] (Fig 3). The arrangement in tandem led to the hypothesis that *fshr* and *lhcgr* arose from a local, small-scale duplication of an ancestral gene, rather than from the whole-genome duplication events that occurred in early vertebrates (2R) [52]. This tandem position was shown to be conserved in all tetrapods [21–52–62]. In the present study, we observed a similar tandem position for *fshr* and *lhcgr* in a basal sarcopterygian, the coelacanth (on scaffold JH127072) (S3 Table).

**Fig 4:**
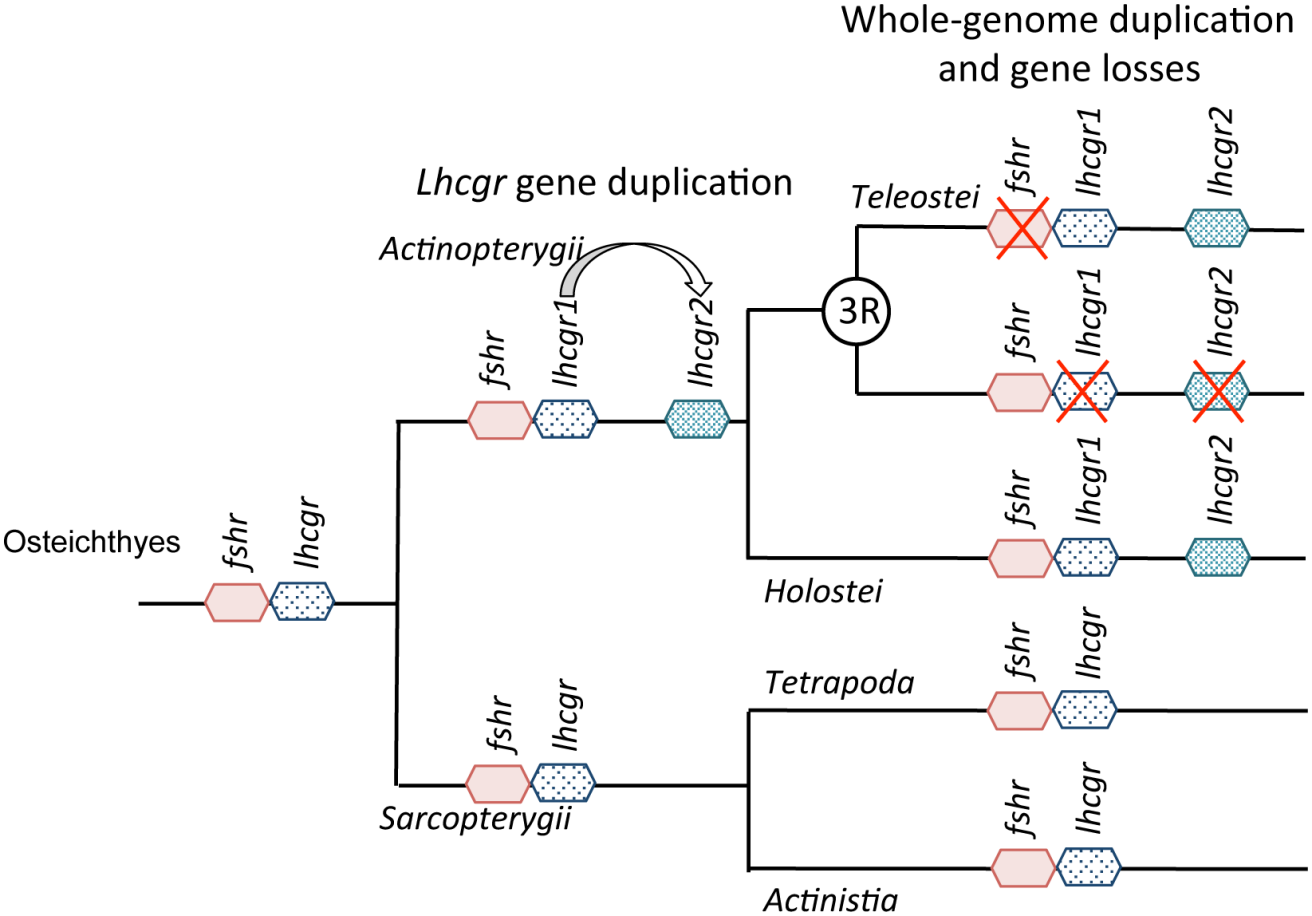
Origin and evolution of duplicated *lhcgr* in actinopterygians. *Fshr*-*lhcgr* tandem was inherited by actinopterygians and sarcopterygians from a commun osteichthyan ancestor. The *fshr*-*lhcgr* tandem was conserved in sarcopterygians including tetrapod [52] and actinistian (coelacanth) lineages. In actinopterygians, a gene duplication event occurred before the emergence of teleosts, leading to two *lhcgr* genes, one maintained in tandem position with *fshr*, and named *lhcgr1*, and the other one named *lhcgr2*. The tandem *fshr*- *lhcgr1* and the *lhcgr2* were conserved on the same chromosome after the emergence of the holostean (spotted gar) lineage. Teleost whole-genome duplication (3R) potentially generated duplicated *fshr*, *lhcgr1* and *lhcgr2*. Early after the 3R, multiple and selective gene losses led to the conservation of only single genes for *fshr*, *lhcgr1* and *lhcgr2*, and to the separation of the *fshr*-*lhcgr(1)* tandem. Additional loss events of *lhcgr1* or *lhcgr2* have occurred independently throughout teleost radiation, so that some extant teleosts have conserved only a single *lhcgr*, *lhcgr1* or *lhcgr2*, according to species.

In the spotted gar genome, *fshr* and duplicated *lhcgr* genes were localized on the same chromosome LG16. We named the spotted gar duplicate *lhcgr* genes according to their genomic position: the *lhcgr* positioned in tandem with *fshr*, as in the sarcopterygians, was called *lhcgr1*, while the other *lhcgr*, separated by around 47 genes (3Mb) from the *fshr*- *lhcgr1* tandem, was called *lhcgr2* (Fig 3). *Lhcgr2* was in a specific genomic region, sharing no paralogs with the genes neighbouring *fshr*-*lhcgr1* tandem. The presence of the *fshr*-*lhcgr(1)* tandem in an actinopterygian as in sarcopterygians, suggests that the local duplication event that gave rise to the tandem, would have occurred early in the gnathostome lineage. We hypothesize that a second local duplication occurred specifically in the actinopterygian lineage, producing the second *lhcgr* (*lhcgr2*) (Fig 4).

Synteny analysis indicated that the genomic region of the three actinopterygian gonadotropin receptors has been duplicated in teleosts, likely as a result of the teleost 3R (Fig 3). Conserved 3R-duplicated genes in this region include for instance *thbs2*, *psme4*, *nrxn1*, *fbxo11*, *prcke*, *ppm1* and *prph2* (Fig 3). Synteny analysis confirmed that despite the 3R, teleost genomes contained no additional gonadotropin receptor genes as compared to spotted gar, due to selective gene losses. These losses have concerned not only duplicate gonadotropin genes but also some other duplicate neighbouring genes (*e*.*g*. *erlec1*, *msh2*, *ston1*, *kcnk12*, *stf2d1*, *mertk*) (Fig 3). Noticeably, as a result of these gene losses, the *fshr- lhcgr1* typical tandem position was not conserved in extant teleosts, in which *fshr* and *lhcgr1* are located on separate chromosomes (*e*.*g*. in Nile tilapia, medaka) (Fig 3). This is in agreement with previous synteny analysis of teleost gonadotropin receptors [21], and with an early study by Oba et al [53] who first indicated by RFLP analysis that *lhcgr* and *fshr* were not genetically linked in tilapia. Teleost *fshr* conserved some neighbouring genes including at the proximal 3’ position the 3R-duplicated *nrxn1*, and at 5’ position the 3R-duplicated *fbxo11* (Fig 3), in agreeement with previous studies [21].

In Nile tilapia, a species that has conserved the duplicated *lhcgr*, both *lhcgr1* and *lhcgr2* were maintained co-localized on the same chromosome (LG13), as in the spotted gar, and were separated from each other by a distance of 350 kb covering 14 predicted genes. Each *lhcgr* had conserved some of the syntenic neighbouring genes, as compared to the spotted gar. In particular, *lhcgr1* was flanked at the proximal 3’ by the 3R-duplicated *nrxn1* and at 5’ by the single *ston1*, and 3R-duplicated *fbxo11*, while *lhcgr2* at 3’ by the single *sft2d1* and at 5’ by the single *mertk*.

Synteny analysis confirmed that, due to additional losses of *lhcgr* genes, some teleost species, despite the 3R, have conserved even less receptors than the gar, as illustrated by the loss of *lhcgr1* in zebrafish and of *lhcgr2* in medaka (Fig 3). In zebrafish, the syntenic blocks including *sft2d1* at 3’, and *mertk* at 5’, were found flanking the *lhcgr2*, as in Nile tilapia and spotted gar. Similarly, medaka *lhcgr1* conserved typical neighbouring genes such as 3R-duplicated *nrxn1* and single *ston1*, as in Nile tilapia and spotted gar.

The current draft genomes of the European and Japanese eels allowed only a partial reconstruction of *fshr*, *lhcgr1* and *lhcgr2* genomic regions (S6 Fig). *Fshr* conserved synteny included 3R-duplicated *nrxn1* and *fbxo11*, *lhcgr1* synteny included single *ston1*, and *lhcgr2* was surrounded by single *sft2d1* and *mertk*, as in spotted gar and Nile tilapia.

In conclusion, synteny analysis supported the phylogeny analysis, indicating the presence of duplicated *lhcgr* in actinopterygians, and of their origin before teleost emergence, independently of teleost 3R (Fig 4). The synteny analysis revealed that only single genes for *fshr*, *lhcgr1* and *lhcgr2* were maintained after the teleost 3R, subsequent to multiple gene deletions, which also led to the physical separation of the *fshr*- *lhcgr(1)* tandem (Fig 4). As discussed by George et al. [52], *fshr*-*lhcgr* tandem in tetrapods forms, together with *nrxn1*, a highly conserved syntenic block, which might be associated with evolutionary constraint that retain regulatory sequences [52]. In teleosts, *fshr* and *lhcgr1* were separated on different chromosomes, but both conserved a 3R-duplicated flanking *nrxn1* gene. Additional studies on gene expression regulation in teleosts may provide information whether separate *fshr* and *lhcgr1* genes are submitted to independent or parallel expression regulation mechanisms, as well as on the potential role of *nrxn1*. In the same perspective, future studies should investigate how differential regulation of duplicated *lhcgr* in actinopterygians, may contribute to the fine-tuning of LH action.

### Proposed nomenclature for actinopterygian, including teleost, duplicated LH receptor genes

Given our findings that two LH receptor genes coexist in holostean and teleost species, as a result of local gene duplication early in the actinopterygian lineage, we proposed a phylogenetically-founded nomenclature. Based on their orthology with the single sarcopterygian *lhcgr*, and on their origin from a local gene duplication, we classically named the duplicated actinopterygian *lhcgr* genes, *lhcgr1* and *lhcgr2*. According to the synteny analysis in the gar, the duplicate *lhcgr* gene that had conserved the close tandem position with *fshr* (an ancestral feature in osteichthyans) was named *lhcgr1*. The other gar *lhcgr* gene was named *lhcgr2*. In teleosts, the whole genome duplication (3R) would have potentially generated a further doubling of LH receptor genes as compared to the gar. However, due to gene losses early after 3R, and before the emergence of the basal group of Elopomorphs, extant teleost species investigated so far do not possess more *lhcgr* genes than the gar. Furthermore, phylogeny analysis clearly clustered teleost and gar sequences in a single *lhcgr1* clade, and a single *lhcgr2* clade. Thus, based on the origin of duplicated *lhcgr* in actinopterygians before the 3R, and on their molecular phylogeny relationships, the same nomenclature, *lhcgr1*, *lhcgr2*, was applied in teleosts, as in the gar. A specific nomenclature had been previously developed for teleost dual LH receptor isoforms, “*lhcgrba*, *lhcgrbb*”, the first letter “b” designing 3R-paralogon “b” and the second letter “a or b” one or the other isoform [21]. This nomenclature was based on the hypotheses of two mutually exclusive LH receptor isoforms in teleosts, located on the same locus, and resulting from interallelic gene conversion and not from gene duplication [21]. These previous hypotheses are invalidated by the present findings. Our proposed nomenclature is based on the new evidences that two LH receptor genes coexist in actinopterygian species, are not located on the same locus, and result from local gene duplication in the actinopterygian lineage, before the emergence of teleosts. In teleosts, duplicated *lhcgr1* and *lhcgr2* genes correspond to previously designed *lhcgrbb* and *lhcgrba* isoforms, respectively.

### Structure comparison of the duplicated LH receptors with special reference to the eel

Amino acid sequence comparison revealed that European eel LHR1 and LHR2 have largely diverged (49.7% identity), almost as much as compared to FSHR (44.9 and 42.2%, respectively) (Table 1). When comparing European and Japanese eel, a striking conservation was found for receptor orthologs (> 97%). A higher conservation was observed between each eel duplicate LH receptor and its respective orthologs from other actinopterygians, as compared to the duplicate paralogs: identity ranged between 54 and 64.2% for orthologs and between 41.5 and 47.9% for paralogs (Table 1). In order to further analyze the evolution of duplicated LHR, we compared the amino acid sequences of each functional domain (Table 1 and S7 Fig). Comparison of protein domain sequence identity between the duplicate European eel LH receptors showed a high conservation of TMD (68.4%), a lower conservation of ECD (43.6%) and a very low conservation of ICD (16.9%).

#### Divergence in the extracellular domain between the duplicate LHR

Amino acid sequences of ECD, a region mainly involved in ligand binding, revealed some divergence between eel duplicated LHR (43.6% identity). As expected, further divergence was observed between each eel LHR and eel FSHR (34.1 and 32.1%, for LHR1 and LHR2, respectively). Among actinopterygians, ECD sequence identity was slightly higher between LHR orthologs (49.7 to 57.8% for LHR1, 46.5 to 52% for LHR2) than between LHR paralogs (39.4 to 44%). The conservation of the ECD between all actinopterygian LHR paralogs was still higher than between LHR and FSHR (30.1 to 34.5% identity) (Table 1).

N-glycosylation sites could be predicted in the ECD of duplicate eel LHR, 3 for LHR1 and 5 for LHR2 (S7 Fig). These sites included for both LHR, a site on LRR7 common to all glycoprotein hormone receptors [19,63], and a site on cb2 conserved among all LHR, except spotted gar LHR2. A N-glycosylation site on LRR3 appeared specific to actinopterygian LHR2. In human, directed-mutagenesis of potential N-glycosylation sites of gonadotropin receptors, or disruption of glycosylation, demonstrated that glycosylation plays a role in the proper folding of the nascent protein into a mature receptor able of binding hormone and signaling [64–65].

An O-sulfation tyrosine site could be predicted in the hinge region of eel LHR2 but not eel LHR1 (S7 Fig). It corresponds to the sulfated tyrosine in the hinge region of human LHCGR (Tyr^331^), FSHR (Tyr^335^) and TSHR (Tyr^385^), that has been postulated to be a prominent feature, conserved among all the GPHR, and involved in receptor activation [40–41,66–67]. This sulfation tyrosine site was predicted in some but not all teleost LHR2, and in none of teleost LHR1 nor teleost FSHR [68]. The lack of potential sulfation tyrosine site in most teleost gonadotropin receptors suggests alternative receptor activation mechanisms than the one requiring the sulfation of the hinge region, as described in mammals [41].

Amino acid comparison of the ECD revealed residues specific of one or the other duplicate LHR, which may constitute fingerprints of each LHR type (S7 Fig). These fingerprints were more marked in teleosts than in spotted gar, indicating that the duplicate LHR may have further diverged in teleosts. Some of these specific residues could be observed in the LRRD domain (S7 Fig). We also found specific features in the hinge region, the LHR1 showing a conserved motif negatively charged (AFHTWRR), that was deleted in the eel LHR2 and substituted in the other teleost LHR2 (S7 Fig). At the level of the Cb3, eel and other teleost LHR1 possess a proline residue (Pro^359^ for eel LHR1), as sarcopterygian LHR as well as spotted gar LHR1 and LHR2, while proline is substituted by alanine (Ala^360^) in eel LHR2, or by alanine or glutamic acid in other teleost LHR2 (S7 Fig). Minor residue replacements were also observed between eel duplicated LHR, as for instance in the LRR6, the substitution of Glu^160^ in LHR1 by aspartic acid (Asp^161^) in LHR2 (S7 Fig). In human LHCGR, replacement by site directed mutagenesis of the corresponding Glu^154^ by Asp was shown to provoke an alteration of the response intensity [69].

All these variations in ECD sequences suggest that duplicated LHR may have diverged in their binding and activation properties in teleosts. So far functional studies on teleost recombinant gonadotropin receptors, using transiently transfected mammalian cell lines (Cos-7 or HEK), were performed either on FSHR, or on one or the other of the duplicate LHR, depending of the LHR type isolated [29–31,68,70–71]. Data on ligand selectivity for eel LHR receptor are only available for eel LHR1 and not for LHR2. Japanese eel LHR1 is activated by eel recombinant LH (recLHR) and not to by eel recFSH, but can also be cross-activated by heterologous gonadotropins such as human chorionic gonadotropin (hCG), human FSH and trout LH [29–31,68]. Binding specificity toward homologous LH, with no cross-reaction of homologous FSH, has also been reported for LHR in other teleosts, whatever the LHR type: for LHR1 in channel catfish [15], Nile tilapia [68] and medaka [72]; for LHR2 in African catfish [73–74], zebrafish [75], salmon [13–14], European sea bass, *Dicentrarchus labrax* [76], and Senegalese sole, *Solea senegalensis* [21]. This indicates that both duplicate LH receptor types would be specific of LH in teleosts. Further functional studies, using recombinant receptors in species having conserved duplicated LHR, are still required to determine whether the dissimilarities between duplicated LHR ECD may affect ligand binding and receptor activation.

#### Conservation of the transmembrane domain between the duplicate LHR

The amino acid comparison of the TMD showed a high identity between the duplicated eel LHR (68.4%) as well as with FSHR (69.9 and 63.9%) (Table 1). A strong amino acid identity was particularly observed among gnathostome gonadotropin receptors for the transmembrane helices 2, 3, 6 and 7, which include key residues participating to the network involved both in the transition of the inactive toward active receptor conformation and in binding the protein G [77] (S7 Fig). Nevertheless, some specific residue divergences between the duplicate LHR could be also noticed within the TMD (S7 Fig). As discussed above for ECD, these fingerprints in the TMD were more marked in teleosts than in spotted gar, indicating that the TMD of the duplicate LHR have further diverged in teleosts (S7 Fig). The phosphorylation site (TVR) in the il3 of the mammalian FSHR was highly conserved in the other gnathostome FSHR including the eel FSHR (S7 Fig). Noticeably, this phosphorylation site was retrieved in spotted gar LHR1, eel LHR1 and some other teleost LHR1, but not in any LHR2 nor in any sarcopterygian LHR (S7 Fig). In the rat, phosphorylation of FSHR il3 was identified as an important determinant in arrestin association [78–79]. Some differences between duplicate LHR TMD sequences might thus confer differential properties in the regulation of signal transduction.

#### High divergence in the cytosolic tail between the duplicate LHR

The sequence comparison of the ICD showed a very low identity between the duplicated eel LHR (16.9%). A higher conservation was noticed among teleost LHR1 (43.6–49.3% identity) as compared to teleost LHR2 (14.7–32%) (Table 1). Specific signatures for teleost LHR1 included amino acids shared with other gnathostome LHR but not LHR2 (such as R^652^ of eel LHR1), as well amino acids conserved only among actinopterygian LHR1 (Cys^697^) or among teleost LHR1 (AYHIK) (S7 Fig). Nevertheless, some sites were highly conserved among all gonadotropin receptors, such as the characteristic cysteine palmitoylation sites of the ICD of the GPCR (e.g. Cys^643^-Cys^644^ for human LHCGR), which are common to all LGR among GPCR, and involved in receptor internalization and recycling [80–81]. Gonadotropin receptor signaling in mammals is mainly mediated by adenylyl cyclase /cAMP pathway, but alternative signaling, such as phospholipase C/inositol phosphate (IP) and beta-arrestin pathways, are also requested for global activation of the response [82]. To our knowledge, investigations on teleost recombinant gonadotropin receptors mainly focused on the cAMP pathway, while a single study also addressed the IP pathway [73]. The large dissimilarity in cytosolic tail sequences between the duplicated LHR may reflect divergence in downstream intracellular signaling cascade and/or receptor internalization and desensitization process. Such differences may represent selective forces that have contributed to the conservation of the duplicated LHR. There are still limited data on the characterization of the signaling pathway activation and receptor trafficking in teleost fishes. Future investigations on the duplicated LH receptors are needed to characterize their respective activation specificities, and further infer the functional evolution of the LHR.

#### Comparative tissue distribution of the three eel gonadotropin receptors

Development of specific qPCR for eel *fshr* and two *lhcgr* allowed analyzing their tissue distribution in European silver eels.

**Fig 5:**
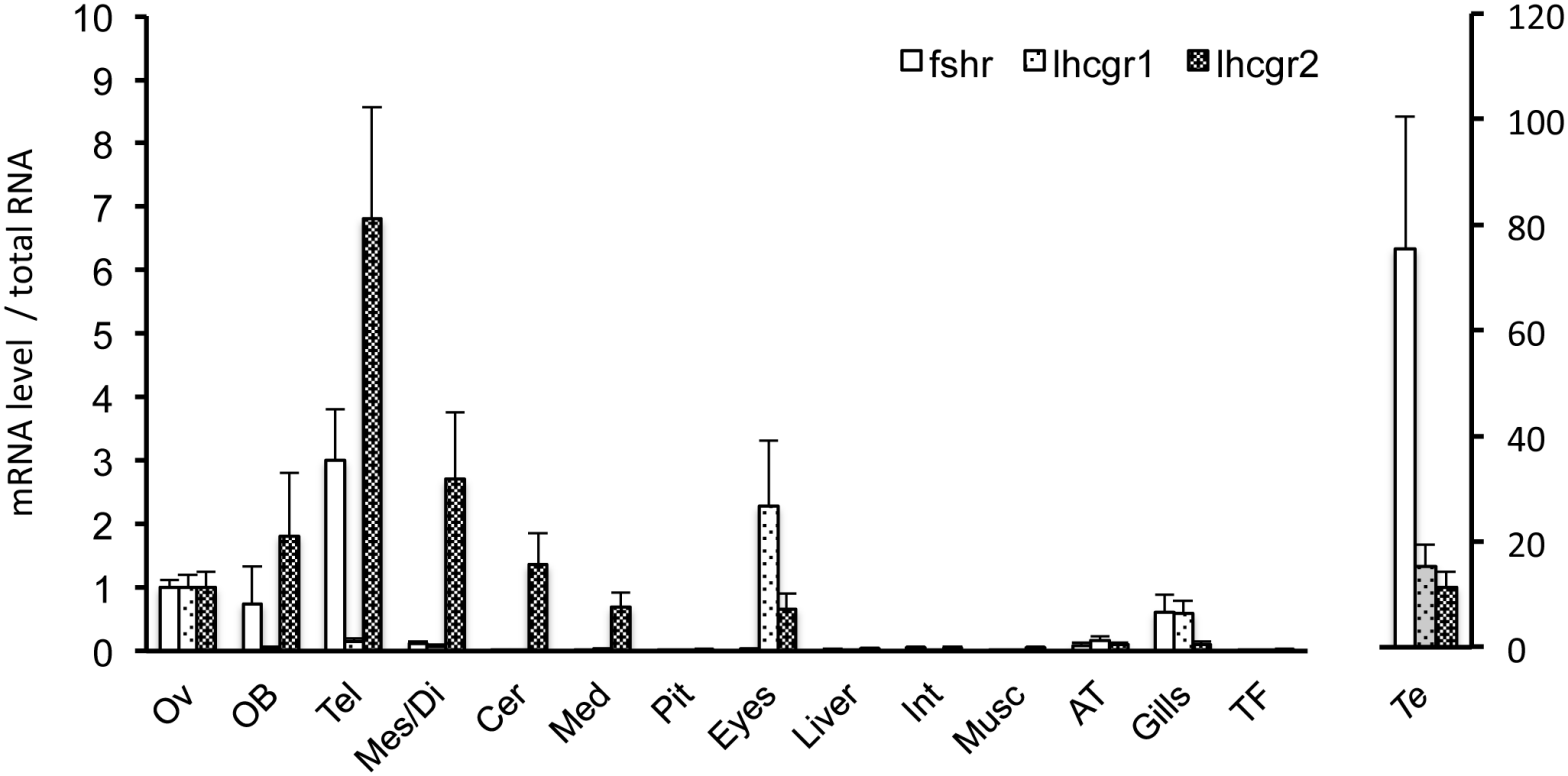
Tissue distribution of *fshr*, *lhcgr1* and *lhcgr2* transcripts in European eel. Messenger RNA levels for *fshr* (B), *lhcgr1* (C) and *lhcgr2* (D) were assayed by qPCR in various tissues of silver female European eels: Ovary (Ov); brain dissected in five parts: olfactory bulbs (Ob) telencephalon (Tel), di- and mes-encephalon (Di/Mes), corpus cerebellum (Cb), and medulla oblongata (Mo); pituitary (Pit); eyes; liver (Liv); intestine (Int); muscle (Mus); adipose tissue (AT); gills and thyroid follicles (TF). Receptor mRNA levels in testis of silver male European eels were also assayed. Data are normalized to total RNA and the expression level in the ovary was set as 1. Means are given ± SEM (n = 8 eels).

Eel *fshr*, *lhcgr1* and *lhcgr2* transcripts are all expressed in the gonads. The three gonadotropin receptors were easily detectable in the immature ovary of female silver eels. The mean ovarian transcript level was used as a reference for comparison with the other tissues (Fig 5). The three receptors were also well detectable in immature testis of male silver eels, with a much higher expression of *fshr* in testis as compared to ovary. Previous qPCR data in Japanese eel also indicated the expression of *fshr* and *lhcgr* in both ovary and testis [27–28,30]. Early radioreceptor and autoradiographic studies in the European eel ovary, revealed binding sites for carp LH and hCG in both granulosa and external theca layers [83]. In Japanese eel, FSHR was immunolocalized in the testis, in both Sertoli cells surrounding spermatogonia cysts and interstitial Leydig cells [28].

In other female teleosts, *in situ* hybridization (ISH) revealed that *fshr* transcript is strongly expressed in granulosa cells, and weakly in theca cells, in chub mackerel, *Scomber japonicus* [84], Atlantic halibut, *Hippoglossus hippoglossus* [85] and Atlantic salmon [86]. *Lhcgr1* transcript is also expressed in granulosa and weakly in theca cells in chub mackerel [84] and [85] Atlantic halibut. Similarly *lhcgr2* transcript is highly expressed in granulosa cells and weakly in theca cells in Atlantic salmon [86]. In medaka, immunocytochemistry (ICC) revealed LHR1 protein labeling in granulosa and theca cells [72].

In male teleosts, *fshr* transcript is localized by ISH in Sertoli cells, and a weak expression is also detected in Leydig cells in African catfish [87]. ISH and ICC studies also showed FSH expression in both Leydig and Sertoli cells in Senegalese sole [21,88]. *Lhcgr2* is expressed in Leydig cells in African catfish [87], and in both Sertoli cells and Leydig cells in zebrafish [89]. In Senegalese sole, ISH and ICC revealed *lhcgr2* transcript and LHR2 in Leydig cells and free spermatids, supporting a direct role of LH on spermiogenesis [21,88,90].

In tetrapods, as in the other vertebrates, *fshr* and *lhcgr* are mainly expressed in the gonads [52]. The present study supports that *fshr* as well as duplicated *lhcgr1* and *lhcgr2* have all conserved a role in the control of gonadal function in the eel. Future studies, including ISH, should aim at further analyzing the cellular localization of the expression of the duplicated *lhcgr* in the eel, as well as in the other fish species, which have also conserved duplicate *lhcgr*.

Eel *fshr*, *lhcgr1* and *lhcgr2* are differentially expressed in the brain (Fig 5). The three gonadotropin receptors are also expressed in non-gonadal tissues, as analyzed by qPCR in the female European silver eel. *Lhcgr2* showed a remarkable expression in the whole brain, higher than the expression measured in the ovary, while *lhcgr1* transcript was at the limit of detection. *Fshr* was highly expressed in some brain regions, such as olfactory bulb and telencephalon (Fig 5). High brain expression of *fshr* was also reported in female Japanese eel at previtellogenic and late vitellogenic stages [30].

*Fshr* transcript is also found in the brain of males or females from other teleost species, such as Atlantic salmon [86], and Atlantic halibut [85]. *Lhcgr2* transcript is expressed in the brain in various teleosts, including African catfish, Atlantic salmon and European sea bass [20,73,86,91]. *Lhcgr1* is also expressed in the brain of the Korean rockfish, *Sebastes schlegelii* [92] and Atlantic halibut [85], while its expression is not detectable in the brain of the channel catfish [15].

*Lhcgr* expression has been reported in the brain of amphibians, birds and mammals including human [93–95]. In mammals, various functions for brain LHR have been suggested such as GnRH regulation [96], sensory modulation, or fetal neurogenesis [97]. In African clawed frog, *Xenopus laevis*, LH controls courtship songs *via* its cognate receptor expressed in forebrain vocal nuclei [95]. To our knowledge there is no report on FSHR in the brain of tetrapods. The fact that *fshr* and *lhcgr* are highly expressed in brain in the eel as in some other teleosts, opens new research avenues on the potential roles of gonadotropin signaling in the vertebrate brain. In the eel, the remarkable differential expression of the duplicated *lhcgr* in brain specifically confers to *lhcgr2* major roles in brain functions.

Eel *fshr*, *lhcgr1 and lhcgr2* are differentially expressed in other non-gonadal tissues. Both *lhcgr1* and *lhcgr2*, but not *fshr*, transcripts were abundant in eye in the eel (Fig 5). *Lhcgr2* transcript has also been reported in eye of the European sea bass and mummichog, *Fundulus heteroclitus* [91,98]. In human, *lhcgr* transcript and LHCGR protein have been identified in retinal photoreceptors, where LH receptor could be involved in local modulation of vision [99]. The expression of *lhcgr1* and *lhcgr2* in eye of the eel may reflect an ancestral role in this organ, which may have been conserved by both duplicated receptors.

*Fshr* and *lhcgr1* were well expressed in the eel gills, while lower levels of *lhcgr2* were found (Fig 5). *Fshr* transcript is also expressed in the gills of Senegalese sole [21], *lhcgr1* in the gills of channel catfish [16] and Atlantic halibut [84], and *lhcgr2* in the gills of Atlantic salmon, European sea bass and mummichog [20,91,98]. This suggests that gonadotropin receptors may participate in the complex multi-endocrine regulation of gill function in teleosts.

*Fshr* as well as both *lhcgr1* and *lhcgr2* were expressed at a low level in the eel adipose tissue. *Lhcgr2* transcript was also reported in the fat tissue of European sea bass [91]. FSHR transcript and protein have been detected in the abdominal fat tissue of female chicken, where FSH stimulates lipid accumulation [100].

Eel *fshr*, *lhcgr1 and lhcgr2* showed a very low expression, at the limit of detection level, in the other tissues investigated, such as pituitary, liver, intestine, muscle and thyroid. *Lhcgr1* transcript is also undetectable by PCR in various non-gonadal tissues, including liver, intestine, heart and spleen, in Korean rockfish [92] and medaka [72]. In contrast, *lhcgr1* transcript has been demonstrated in muscle, liver, stomach and heart in channel catfish [16] and in spleen in goby [101]. *Lhcgr2* transcript has been observed also in liver, muscle and heart in African catfish [73], and in intestine and pituitary in European sea bass and mummichog [91,98]. This suggests variations in the non-gonadal target tissues and roles of LH, according to teleost species.

#### Evolutionary scenario of gonadotropin receptors

**Fig 6:**
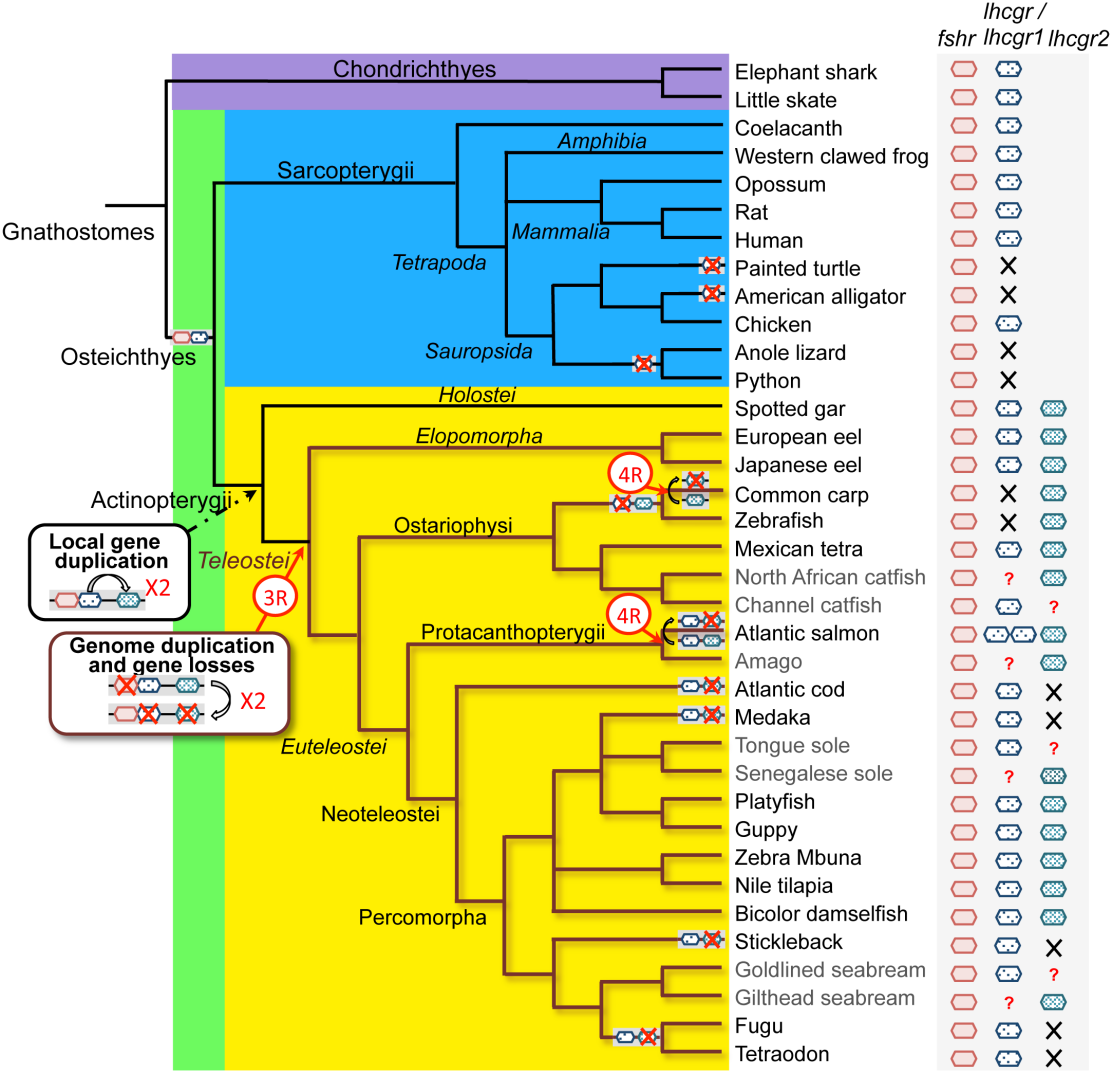
Current status and evolutionary scenario of gonadotropin receptors in gnathostomes. The presence of gonadotropin receptors (*fshr*, *lhcgr*, *lhcgr1*, *lhcgr2*) in representative extant species of gnathostome lineages is shown on the right. In some species, the presence or absence of some gonadotropin receptors could not be assessed, due to the lack of genomic data, and these cases are indicated by a question mark. Two gonadotropin receptors, *fshr* and *lhcgr* are present in gnathostomes including chondrichthyans and osteichthyans. They were positioned in tandem in the early osteichthyes. The *fshr*-*lhcgr* tandem was maintained after the split between sarcopterygians and actinopterygians. Gene loss of *lhcgr* in various sauropsid groups led to the presence of only *fshr* in some extant sauropsids. A duplicated *lhcgr* was generated by a local gene duplication event in the actinopterygian lineage before the emergence of teleosts. Teleost whole-genome duplication (3R) generated duplicated *fshr*, *lhcgr1* and *lhcgr2*. Multiple and selective gene losses after the 3R led to the maintenance of only a single copy of *fshr*, *lhcgr1* and *lhcgr2* and to the physical separation of the tandem *fshr*-*lhcgr*. Additional *lhr* losses occurred independently through the teleost radiation, *i*.*e*. *lhcgr1* was lost in cyprinidae and *lhcgr2* in some percomorphs. Recent tetraploidization (4R) event has generated additional copies of *lhcgr1* in salmonids.

Genome search, phylogeny and synteny analyses allowed us to revise the number and evolutionary history of gonadotropin receptors in gnathostomes (Fig 6). A single copy of *fshr* gene is present in gnathostomes. A single *lhcgr* gene is also present in sarcopterygians, including in the basal representative species, the coelacanth, and it is positioned in tandem with *fshr*. This tandem, also observed in a holostean actinopterygian, the gar (for *fshr-lhcgr1*), is likely the result of a local duplication of an ancestral gonadotropin receptor gene in early gnathostomes (Fig 6).

In the elephant shark, *fshr* and *lhcgr* were not found positioned in tandem but each gene was on a distinct scaffold (S3 Table). This questions the origin of the *fshr-lhcgr* tandem. This suggests either that the tandem arose only in osteichthyan lineage after the emergence of the chondrichthyans, or that the tandem position was not maintained in the extant chondrichthyans. Further analysis using other chondrichthyan genomes would allow a better resolution of the glycoprotein hormone receptor evolution history.

Among tetrapods, genome search in sauropsids revealed multiple and independent *lhcgr* gene mutation or loss events in the squamate, crocodilian, and chelonian lineages. This suggests that gonadotropin action would be mediated only by *fshr* in these species. In contrast, birds have conserved *lhcgr* and *fshr*, positioned in tandem, as in the other tetrapods (Fig 3).

Our investigation revealed that duplicated *lhcgr* (named *lhcgr1* and *lhcgr2*) are present in some actinopterygians, including the eel and some other teleosts, as well as in a holostean species, the gar. The coexistence of duplicated *lhcgr* in various actinopterygians, including extant teleosts, rules out a former hypothesis, that the duality of teleost receptors would result interallelic gene conversion and not from gene duplication [21]. We raised the hypothesis that these duplicated *lhcgr* genes may be the result of a second local gene duplication event, that would have occurred early in the actinopterygian lineage, before the split between holosteans and teleosts (Fig 6).

These duplicated *lhcgr*, which are present in a non-teleost actinopterygian, the gar, are clearly not the result of the teleost-specific whole-genome duplication (3R). Furthermore, phylogeny and synteny analyses show that there was no impact of 3R on the number of gonadotropin receptor genes in extant teleosts, due to multiple gene losses after the 3R. Our investigation in the eel, a representative species of a basal group of teleosts (elopomorph) suggests that these losses of 3R-duplicated gonadotropin receptor genes would have occurred soon after the 3R. Thus, only one copy of the 3R-duplicated *fshr*, *lhcgr1* and *lhcgr2* was retained, while the other one was lost early during teleost radiation (Fig 6), likely *via* the rediploidization process that follows polyploidization event. In agreement with previous studies [21,53], one consequence of these 3R-duplicate gonadotropin gene losses in teleosts, is the separation of the *fshr-lhcgr* tandem in all extant teleosts, with potential implications in the regulatory mechanisms of their expression.

Additional loss events of *lhcgr1* or *lhcgr2* have occurred independently throughout teleost radiation, so that some extant teleosts have conserved only a single *lhcgr* (*lhcgr1* or *lhcgr2)*, according to species (Fig 6). The loss of *lhcgr1* in cyprinids and the preferential loss of *lhcgr2* that occurred repeatedly through the percomorph lineage, suggest that the presence of two *lhcgr* may not be fully stabilized in teleosts. This diversity in *lhcgr* duplicate gene conservation or losses among teleosts, also raises the question of the possible presence of duplicate receptors in teleost species for which only one *lhcgr* has been characterized, while genomic data are still limited.

The recent tetraploidization events (4R) that occurred independently in cyprinids and salmonids, are reflected by some additional numbers of *lhcgr* genes, such as in Atlantic salmon (two *lhcgr1* genes) (Fig 6). In contrast, loss of the other putative 4R-duplicated gonadotropin receptors in these species, illustrates the progressive gene fractionation occurring in polyploid genome [55].

Comparisons between gonadotropin receptor amino acid sequences revealed some divergences between teleost LHR1 and LHR2 paralogs, mainly concerning the ECD and ICD domains. These differences, which may affect receptor-binding properties, signaling pathways and recycling, might represent selective forces that have contributed to the conservation of duplicate LHR. We also compared the tissue distribution of the expression of *fshr* and duplicated *lhcgr* transcripts, using the eel as a model. All three gonadotropin receptors are expressed in the ovary and testis, and are thus involved in the mediation of the control of male and female reproductive function. In non-gonadal tissues, differential expression patterns were revealed. For instance, high expression of *fshr* and *lhcgr2*, but very low *lhcgr1* expression was observed in the brain. Different gene regulatory environments could have driven the duplicated *lhcgr* to distinct extra-gonadal functions. This may act as evolutionary forces in the conservation of the duplicated *lhcgr*. The present discovery of duplicated *lhcgr* in actinopterygians, including various teleost species of biological, ecological and aquaculture relevance, opens new research avenues in basic and applied reproductive endocrinology, as well as in evolutionary biology. It will promote future investigation on the comparative function and regulation of gonadotropin receptors, in order to decipher the physiological significance of the conservation of duplicated LHR receptors in various species.

## Supporting Information

S1 Fig Annotation of *fshr*, *lhcgr1* and *lhcgr2* CDS from contig and scaffold sequences of European eel genome assembly. (TIF)

S2 Fig Nucleotide and amino acid sequence of the European eel FSHR. Nucleotide and deduced amino acid sequence of the eel fshr CDS. Numbers on the left refer to position of the nucleotide residues (top) and the amino acid (bottom). The predicted signal peptide is indicated in bold italics. Cysteine residues are indicated by red boxes. Putative sites for N-linked glycosylation are indicated by grey boxes. The eleven β-strand motifs of the LRR, identified by Pfam Blast and sequence alignment with the human FSHR, are indicated in blue light boxes. The position of the seven predicted alpha-helices is shown as yellow boxes. Potential sites for PKC phosphorylation are indicated by orange boxes.
(TIF)

S3 Fig Nucleotide and amino acid sequence of the European eel LHR1. Nucleotide and deduced amino acid sequence of the eel *lhcgr1* CDS. For symbols see legend of S3 Fig.
(TIF)

S4 Fig Nucleotide and amino acid sequence of the European eel LHR2. Nucleotide and deduced amino acid sequence of the eel *lhcgr2* CDS. For symbols see legend of S3 Fig.
(TIF)

S5 Fig Annotation of *fshr*, *lhcgr1* and *lhcgr2* CDS from contig and scaffold sequences of Atlantic salmon genome assembly. (TIF)

S6 Fig Reconstructed genomic syntenic regions of eel f*shr*, *lhcgr1* and *lhcgr2*. (TIF)

S7 Fig Amino acid sequence alignment of gonadotropin receptors. Sequences of LHR and FSHR of representative vertebrate species, including chondrichthyan sarcopterygians and actinopterygians, were aligned using ClustalW and manually adjusted. Cysteine residues are in bold red. Cysteine residues potentially involved in a disulfide bond are highlighted in blue. Orange residues represent conserved residues (> 85%) among FSHR and LHR as determined from a general alignment including additional sequences (56 FSHR and 69 LHR sequences, S1 Table). Signal peptides are indicated in italics. Leucine-rich repeats (LRR) of the extracellular domain are delimited by a horizontal black line, and beta-strands of the LRR are highlighted in light blue. Conserved transmembrane alpha-helices (TH) of the transmembrane domain are highlighted in grey. Putative N-glycosylation sites are highlighted in green, potential tyrosine sulfation sites in the hinge region are in red, potential phosphorylation sites in pink and palmytoilation site in green. Specific LHR1 amino acid signatures are highlighted in orange while specific LHR2 signatures in yellow. Abbreviations: Cb, cysteine box; el, extracellular loop; il, intracellular loop.
(TIF)

S1 Table References of gonadotropin receptor sequences. (XLSX)

S2 Table Primer sets for cloning and quantitative real-time PCR of European eel gonadotropin receptors. (DOCX)

S3 Table Database references for flanking genes of *fshr* and *lhcgr* genomic regions. (XLSX)

## References

[1] Pierce J, Parsons TF. Glycoprotein hormones: structure and function. J Annu Rev Biochem. 1981;50: 465–495. 10.1146/annurev.bi.50.070181.002341 6267989

[2] Fox KM, Dias JA, Van Roey P. Three-dimensional structure of human follicle-stimulating hormone. Mol. Endocrinol. 2001;15: 378–389. 11222739 10.1210/mend.15.3.0603

[3] Quérat B, Tonnerre-Doncarli C, Géniès F, Salmon C. Duality of gonadotropins in gnathostomes. Gen Comp Endocrinol. 2001;124: 308–314. 11742514 10.1006/gcen.2001.7715

[4] McFarland KC, Sprengel R, Phillips HS, Köhler M, Rosemblit N, Nikolics K, et al Lutropin-choriogonadotropin receptor: an unusual member of the G protein-coupled receptor family. Science. 1989;245: 494–499. 2502842 10.1126/science.2502842

[5] Sprengel R, Braun T, Nikolics K, Segaloff DL, Seeburg PH. The testicular receptor for follicle stimulating hormone: structure and functional expression of cloned cDNA. Mol Endocrinol.1990;4: 525–530. 2126341 10.1210/mend-4-4-525

[6] Loosfelt H, Misrahi M, Atger M, Salesse R, Vu Hai-Luu Thi MT, Jolivet A, et al Cloning and sequencing of porcine LH-hCG receptor cDNA: variants lacking transmembrane domain. Science. 1989;245: 525–528. 2502844 10.1126/science.2502844

[7] Minegishi T, Nakamura K, Takakura Y, Miyamoto K, Hasegawa Y, Ibuki Y, et al Cloning and sequencing of human LH/hCG receptor cDNA. *Biochem*. *Bio phys*. *Res*. *Commun*. 1990;172: 1049–1054. 10.1016/0006-291x(90)91552-4 2244890

[8] Minegishi T, Nakamura K, Takakura Y, Ibuki Y, Igarashi M. Cloning and sequencing of human FSH receptor cDNA. Biochem. Biophys. Res. Commun. 1991;175: 1125–1130. 1709010 10.1016/0006-291x(91)91682-3

[9] Remy JJ, Lahbib-Mansais Y, Yerle M, Bozon V, Couture L, Pajot E, et al The porcine follitropin receptor: cDNA cloning, functional expression and chromosomal localization of the gene. Gene 1995;163: 257–261. 7590277 10.1016/0378-1119(95)00385-j

[10] Johnson AL, Bridgham JT, Wagner B. Characterization of a chicken luteinizing hormone receptor (cLH-R) complementary deoxyribonucleic acid, and expression of cLH-R messenger ribonucleic acid in the ovary. Biol Reprod. 1996;55: 304–309. 8828833 10.1095/biolreprod55.2.304

[11] Wakabayashi N., Suzuki A., Hoshino H., Nishimori K., Mizuno S. The cDNA cloning and transient expression of a chicken gene encoding a follicle-stimuating hormone receptor. Gene 1997;197: 121–127. 9332357 10.1016/s0378-1119(97)00250-3

[12] Oba Y, Hirai T, Yoshiura Y, Yoshikuni M, Kawauchi H, Nagahama Y. Cloning, functional characterization, and expression of a gonadotropin receptor cDNA in the ovary and testis of amago salmon (*Oncorhynchus rhodurus*). Biochem Biophys Res Commun. 1999;263: 584–590. 10491336 10.1006/bbrc.1999.1346

[13] Oba Y, Hirai T, Yoshiura Y, Yoshikuni M, Kawauchi H, Nagahama Y. The duality of fish gonadotropin receptors: cloning and functional characterization of a second gonadotropin receptor cDNA expressed in the ovary and testis of amago salmon (*Oncorhynchus rhodurus*). Biochem Biophys Res Commun. 1999;265: 366–371. 10558873 10.1006/bbrc.1999.1700

[14] Bogerd J, Blomenröhr M, Andersson E, van der Putten HH, Tensen CP, Vischer HF, et al Discrepancy between molecular structure and ligand selectivity of a testicular follicle-stimulating hormone receptor of the African catfish (*Clarias gariepinus*). Biol Reprod. 2001;64: 1633–1643. 11369589 10.1095/biolreprod64.6.1633

[15] Kumar RS, Ijiri S, Trant JM. Molecular biology of channel catfish gonadotropin receptors: 1. Cloning of a functional luteinizing hormone receptor and preovulatory induction of gene expression. Biol Reprod. 2001;64: 1010–1018. 11207219 10.1095/biolreprod64.3.1010

[16] Kumar RS, Ijiri S, Trant JM. Molecular biology of the channel catfish gonadotropin receptors: 2. Complementary DNA cloning, functional expression, and seasonal gene expression of the follicle-stimulating hormone receptor. Biol Reprod. 2001;65: 710–717. 11514332 10.1095/biolreprod65.3.710

[17] Urbatzka R1, Lorenz C, Lutz I, Kloas W. Expression profiles of LHbeta, FSHbeta and their gonadal receptor mRNAs during sexual differentiation of *Xenopus laevis* tadpoles. Gen Comp Endocrinol. 2010;168: 239–244. 10.1016/j.ygcen.2010.02.012 20171219

[18] Suda M, Kodama M, Oshima Y, Yamamoto K, Nakamura Y, Tanaka S, et al Up-regulation of FSHR expression during gonadal sex determination in the frog *Rana rugosa* . Gen Comp Endocrinol. 2011;172: 475–486. 10.1016/j.ygcen.2011.04.011 21521644

[19] Levavi-Sivan B, Bogerd J, Mañanos EL, Gomez A, Lareyre JJ. Perspectives on fish gonadotropins. Gen Comp Endocrinol. 2010;165: 412–437. 10.1016/j.ygcen.2009.07.019 19686749

[20] Maugars G, Schmitz M. Molecular cloning and characterization of FSH and LH receptors in Atlantic salmon (*Salmo salar* L). Gen Comp Endocrinol. 2006;149: 108–117. 16764877 10.1016/j.ygcen.2006.04.011

[21] Chauvigné F, Tingaud-Sequeira A, Agulleiro MJ, Calusinska M, Gómez A, Finn RN, et al Functional and evolutionary analysis of flatfish gonadotropin receptors reveals cladal- and lineage-level divergence of the teleost glycoprotein receptor family. Biol Reprod. 82: 1088–1102. 2010;109.082289. 20200210 10.1095/biolreprod.109.082289

[22] Hoegg S, Brinkmann H, Taylor JS, Meyer A. Phylogenetic timing of the fish-specific genome duplication correlates with the diversification of teleost fish. J Mol Evol. 2004;59: 190–203. 15486693 10.1007/s00239-004-2613-z

[23] Chen JN, López JA, Lavoué S, Miya M, Chen WJ. Phylogeny of the Elopomorpha (Teleostei): evidence from six nuclear and mitochondrial markers. Mol Phylogenet Evol. 2014;70: 152–161. 10.1016/j.ympev.2013.09.002 24041936

[24] Henkel CV, Burgerhout E, de Wijze DL, Dirks RP, Minegishi Y, Jansen HJ, et al Primitive duplicate hox clusters in the European eel's genome. PloS One 2012;7: e32231 10.1371/journal.pone.0032231 22384188 PMC3286462

[25] Henkel CV, Dirks RP, de Wijze DL, Minegishi Y, Aoyama J, Jansen HJ, et al First draft genome sequence of the Japanese eel, *Anguilla japonica* . Gene 2012;511: 195–201. 10.1016/j.gene.2012.09.064 23026207

[26] Pasquier J, Lafont AG, Jeng SR, Morini M, Dirks R, van den Thillart G, et al Multiple kisspeptin receptors in early osteichthyans provide new insights into the evolution of this receptor family. PloS One 2012;7: e48931 10.1371/journal.pone.0048931 23185286 PMC3502363

[27] Jeng SR, Yueh WS, Chen GR, Lee YH, Dufour S, Chang CF. Differential expression and regulation of gonadotropins and their receptors in the Japanese eel, *Anguilla japonica* . Gen Comp Endocrinol. 2007;154: 161–173. 17597622 10.1016/j.ygcen.2007.05.026

[28] Ohta T, Miyake H, Miura C, Kamei H, Aida K, Miura T. Follicle-stimulating hormone induces spermatogenesis mediated by androgen production in Japanese eel, *Anguilla japonica* . Biol Reprod. 2007; 77: 970–977. 17761645 10.1095/biolreprod.107.062299

[29] Kazeto Y, Kohara M, Miura T, Miura C, Yamaguchi S, Trant JM, et al Japanese eel follicle-stimulating hormone (Fsh) and luteinizing hormone (Lh): production of biologically active recombinant Fsh and Lh by Drosophila S2 cells and their differential actions on the reproductive biology. Biol Reprod. 2008;79: 938–946. 10.1095/biolreprod.108.070052 18685126

[30] Kazeto Y, Kohara M, Tosaka R, Gen K, Yokoyama M, Miura C, et al Molecular characterization and gene expression of Japanese eel (Anguilla japonica) gonadotropin receptors. Zoolog Sci. 2012;29: 204–211. 10.2108/zsj.29.204 22379989

[31] Minegishi Y, Dirks RP, de Wijze DL, Brittijn SA, Burgerhout E, Spaink HP, et al Quantitative bioassays for measuring biologically functional gonadotropins based on eel gonadotropic receptors. Gen Comp Endocrinol. 2012;178: 145–152. 10.1016/j.ygcen.2012.04.030 22580328

[32] Aroua S, Maugars G, Jeng SR, Weltzien FA, Rousseau K, Dufour S. Pituitary gonadotropins FSH and LH are oppositely regulated by the activin/follistatin system in a basal teleost, the eel. Gen Comp Endocrinol. 2012;175: 82–91. 10.1016/j.ygcen.2011.10.002 22019479

[33] Maugars G, Dufour S, Cohen-Tannoudji J, Quérat B. Multiple thyrotropin β-subunit and thyrotropin receptor-related genes arose during vertebrate evolution. PloS One. 2014;9: e111361 10.1371/journal.pone.0111361 25386660 PMC4227674

[34] Altschul SF, Gish W, Miller W, Myers EW, Lipman DJ. Basic local alignment search tool. J Mol Biol. 1990; 215: 403–410. 10.1016/s0022-2836(05)80360-2. http://blast.ncbi.nlm.nih.gov/Blast.cgi. 2231712

[35] Koressaar T, Remm M. Enhancements and modifications of primer design program Primer3. Bioinformatics 2007;23: 1289–1291. 10.1093/bioinformatics/btm091. http://primer3.ut.ee. 17379693

[36] Petersen T N, Brunak S, von Heijne G, Nielsen H. SignalP 4.0: discriminating signal peptides from transmembrane regions. Nature Methods 2011;8: 785–786. http://www.cbs.dtu.dk/services/SignalP/. 10.1038/nmeth.1701 21959131

[37] Bernsel A, Viklund H, Hennerdal A, Elofsson A. TOPCONS: consensus prediction of membrane protein topology. *Nucleic Acids Res*. 2009;37: 465–468. http://topcons.cbr.su.se. 10.1093/nar/gkp363 PMC2703981 19429891

[38] De Castro E, Sigrist CJA, Gattiker A, Bulliard V, Langendijk-Genevaux PS, Gasteiger E, et al ScanProsite: detection of PROSITE signature matches and ProRule-associated functional and structural residues in proteins. Nucleic Acids Res. 2006;34: 362–365. http://prosite.expasy.org. 10.1093/nar/gkl124 PMC1538847 16845026

[39] Finn RD, Bateman A, Clements J, Coggill P, Eberhardt RY, Eddy SR, et al The Pfam protein families database. Nucleic Acids Res. 2014;42: 222–230. http://pfam.xfam.org. 10.1093/nar/gkt1223 PMC3965110 24288371

[40] Jiang X, Liu H, Chen X, Chen PH, Fischer D, Sriraman V, et al Structure of follicle-stimulating hormone in complex with the entire ectodomain of its receptor. Proc Natl Acad Sci U S A 2012;109: 12491–12496. 10.1073/pnas.1206643109 22802634 PMC3411987

[41] Jiang X, Dias JA, He X. Structural biology of glycoprotein hormones and their receptors: insights to signaling. Mol Cell Endocrinol. 2014;382: 424–451. 10.1016/j.mce.2013.08.021 24001578

[42] Chang WC, Lee TY, Shien DM, Hsu JBK, Horng JT, Hsu PC, et al "Incorporating support vector machine for identifying protein tyrosine sulfation sites" J Comput Chem. 2009;30: 2526–2537. 10.1002/jcc.21258. http://sulfosite.mbc.nctu.edu.tw. 19373826

[43] Kumari B, Kumar R, Kumar M. PalmPred: An SVM based palmitoylation prediction method using sequence profile information. PloS One 2014;9: e89246 10.1371/journal.pone.0089246. http://14.139.227.92/mkumar/palmpred/ 24586628 PMC3929663

[44] Guindon S, Dufayard JF, Lefort V, Anisimova M, Hordijk W, Gascuel O. New Algorithms and Methods to Estimate Maximum-Likelihood Phylogenies: Assessing the Performance of PhyML 3.0 Syst Biol. 2010;59: 307–321. 10.1093/sysbio/syq010 20525638

[45] Galtier N, Gouy M, Gautier C. SeaView and Phylo_win, two graphic tools for sequence alignment and molecular phylogeny. Comput Applic Biosci. 1996:12: 543–548. 10.1093/bioinformatics/12.6.543 9021275

[46] Louis A, Muffato M, Roest Crollius H. Genomicus: five genome browsers for comparative genomics in eukaryota; Nucleic Acids Res. 2013;41: 700–705. 10.1093/nar/gks1156 PMC3531091 23193262

[47] Kobe B, Kajava AV. The leucine-rich repeat as a protein recognition motif. Curr Opin Struct Biol. 2001;11: 725–732. 11751054 10.1016/s0959-440x(01)00266-4

[48] Smits G, Campillo M, Govaerts C, Janssens V, Richter C, Vassart G, et al Glycoprotein hormone receptors: determinants in leucine-rich repeats responsible for ligand specificity. EMBO J. 2003;22: 2692–2703. 12773385 10.1093/emboj/cdg260 PMC156757

[49] Moyle WR, Xing Y, Lin W, Cao D, Myers RV, Kerrigan JE, et al Model of glycoprotein hormone receptor ligand binding and signaling. *J*. *Biol*. *Chem*. 2004;279: 44442–44459. 10.1074/jbc.m406948200 15304493

[50] Fan QR, Hendrickson WA. Assembly and structural characterization of an authentic complex between human follicle stimulating hormone and a hormone-binding ectodomain of its receptor. Mol Cell Endocrinol. 2007;260: 73–82. 17045735 10.1016/j.mce.2005.12.055 PMC2012943

[51] Ulloa-Aguirre A, Zariñán T, Pasapera AM, Casas-González P, Dias JA. Multiple facets of follicle-stimulating hormone receptor function. Endocrine. 2007;32: 251–263. 10.1007/s12020-008-9041-6 18246451

[52] George JW, Dille EA, Heckert LL. Current concepts of follicle-stimulating hormone receptor gene regulation. Biol Reprod. 2011;84: 7–17. 10.1095/biolreprod.110.085043 20739665 PMC4480823

[53] Oba Y, Hirai T, Yoshiura Y, Kobayashi T, Nagahama Y. Fish gonadotropin and thyrotropin receptors: the evolution of glycoprotein hormone receptors in vertebrates. Comp Biochem Physiol B Biochem Mol Biol. 2001;129: 441–448. 11399478 10.1016/s1096-4959(01)00374-8

[54] Troppmann B, Kleinau G, Krause G, Gromoll J. Structural and functional plasticity of the luteinizing hormone/choriogonadotrophin receptor. Hum Reprod. 2013;19: 583–602. 10.1093/humupd/dmt023 23686864

[55] Berthelot C, Brunet F, Chalopin D, Juanchich A, Bernard M, Noël B, et al The rainbow trout genome provides novel insights into evolution after whole-genome duplication in vertebrates. Nat Commun. 2014; 5:3657 10.1038/ncomms4657 24755649 PMC4071752

[56] Henkel CV, Dirks RP, Jansen HJ, Forlenza M, Wiegertjes GF, Howe K, et al Comparison of the exomes of common carp (*Cyprinus carpio*) and zebrafish (*Danio rerio*). Zebrafish. 2012;9: 59–67. 10.1089/zeb.2012.0773 22715948 PMC3371262

[57] Xu P, Zhang X, Wang X, Li J, Liu G, Kuang Y, et al Genome sequence and genetic diversity of the common carp, *Cyprinus carpio* . Nat Genet. 2014;46: 1212–1219. 10.1038/ng.3098 25240282

[58] Chiari Y1, Cahais V, Galtier N, Delsuc F. Phylogenomic analyses support the position of turtles as the sister group of birds and crocodiles (Archosauria). BMC Biol. 2012;10: 65 10.1186/1741-7007-10-65 22839781 PMC3473239

[59] Shaffer HB, Minx P, Warren DE, Shedlock AM, Thomson RC, Valenzuela N, et al The western painted turtle genome, a model for the evolution of extreme physiological adaptations in a slowly evolving lineage. Genome Biol. 2013;14: R28 10.1186/gb-2013-14-3-r28 23537068 PMC4054807

[60] Pasquier J, Lafont AG, Rousseau K, Quérat B, Chemineau P, Dufour S. Looking for the bird Kiss: evolutionary scenario in sauropsids. BMC Evol Biol. 2014;14: 30 10.1186/1471-2148-14-30 24552453 PMC4015844

[61] Rousseau-Merck MF, Atger M, Loosfelt H, Milgrom E, Berger R. The chromosomal localization of the human follicle-stimulating hormone receptor gene (FSHR) on 2p21-p16 is similar to that of the luteinizing hormone receptor gene. Genomics. 1993;15: 222–224. 8432542 10.1006/geno.1993.1041

[62] Montgomery GW, Tate ML, Henry HM, Penty JM, Rohan RM. The follicle-stimulating hormone receptor and luteinizing hormone receptor genes are closely linked in sheep and deer. J Mol Endocrinol. 1995;15: 259–265. 8748132 10.1677/jme.0.0150259

[63] Kumar RS, Trant JM. Piscine glycoprotein hormone (gonadotropin and thyrotropin) receptors: a review of recent developments. Comp Biochem Physiol B Biochem Mol Biol. 2001;129: 347–355. 11399468 10.1016/s1096-4959(01)00317-7

[64] Davis D, Li X, Segaloff DL. Identification of the sites of N- linked glycosylation on the follicle-stimulating hormone (FSH) receptor and assessment of their role in FSH receptor function. Mol endorcinol. 1995;9: 159–170. 10.1210/mend.9.2.7776966 7776966

[65] Davis DP, Rozell TG, Liu X, Segaloff DL. The six N-linked carbohydrates of the lutropin/choriogonadotropin receptor are not absolutely required for correct folding, cell surface expression, hormone binding, or signal transduction. Mol Endocrinol. 1997;11: 550–562. 9139799 10.1210/mend.11.5.9927

[66] Bonomi M, Busnelli M, Persani L, Vassart G, Costagliola S. Structural differences in the hinge region of the glycoprotein hormone receptors: evidence from the sulfated tyrosine residues. Mol Endocrinol. 2006;20: 3351–3363. 16901970 10.1210/me.2005-0521

[67] Costagliola S, Panneels V, Bonomi M, Koch J, Many MC, Smits G, et al Tyrosine sulfation is required for agonist recognition by glycoprotein hormone receptors. EMBO J. 2002;21: 504–513. 11847099 10.1093/emboj/21.4.504 PMC125869

[68] Aizen J, Kowalsman N, Kobayashi M, Hollander L, Sohn YC, Yoshizaki G, et al Experimental and computational study of inter- and intra- species specificity of gonadotropins for various gonadotropin receptors. Mol. Cell. Endocrinol. 2012;364: 89–100. 10.1016/j.mce.2012.08.013 22954681

[69] Angelova K, de Jonge H, Granneman JC, Puett D, Bogerd J. Functional differences of invariant and highly conserved residues in the extracellular domain of the glycoprotein hormone receptors. J Biol Chem. 2010;285: 34813–34827. 10.1074/jbc.M110.148221 20736161 PMC2966097

[70] Sambroni E, Le Gac F, Breton B, Lareyre JJ. Functional specificity of the rainbow trout (*Oncorhynchus mykiss*) gonadotropin receptors as assayed in a mammalian cell line. J Endocrinol. 2007;195: 213–228. 17951533 10.1677/JOE-06-0122

[71] Saha A, Sambroni E, Bogerd J, Schulz RW, Le Gac F, Lareyre J-J. The cell context influences rainbow trout gonadotropin receptors selectivity. Indian J. Sci. Technol., 9th ISRPF 2011;4: 8 ISSN: 0974- 6846.

[72] Ogiwara K, Fujimori C, Rajapakse S, Takahashi T. Characterization of luteinizing hormone and luteinizing hormone receptor and their Indispensable role in the ovulatory process of the medaka. PloS One 2013;8: e54482 10.1371/journal.pone.0054482 23372734 PMC3553140

[73] Vischer HF, Bogerd J. Cloning and functional characterization of a gonadal luteinizing hormone receptor complementary DNA from the African catfish (*Clarias gariepinus*). Biol Reprod. 2003;68: 262–271. 12493722 10.1095/biolreprod.102.004515

[74] Vischer HF, Marques RB, Granneman JC, Linskens MH, Schulz RW, Bogerd J. Receptor-selective determinants in catfish gonadotropin seat-belt loops. Mol Cell Endocrinol. 2004;224: 55–63. 15353180 10.1016/j.mce.2004.06.011

[75] Kwok HF, So WK, Wang Y, Ge W. Zebrafish gonadotropins and their receptors: I. Cloning and characterization of zebrafish follicle-stimulating hormone and luteinizing hormone receptors—evidence for their distinct functions in follicle development. Biol Reprod. 2005;72: 1370–1381. 15728795 10.1095/biolreprod.104.038190

[76] Molés G, Zanuy S, Muñoz I, Crespo B, Martínez I, Mañanós E, et al Receptor specificity and functional comparison of recombinant sea bass (*Dicentrarchus labrax*) gonadotropins (FSH and LH) produced in different host systems. Biol Reprod. 2001;84: 1171–1181. 10.1095/biolreprod.110.086470 21293031

[77] Angelova K, Felline A, Lee M, Patel M, Puett D, Fanelli F. Conserved amino acids participate in the structure networks deputed to intramolecular communication in the lutropin receptor. Cell Mol Life Sci. 2011;68: 1227–1239. 10.1007/s00018-010-0519-z 20835841 PMC11114907

[78] Bhaskaran RS, Min L, Krishnamurthy H, Ascoli M. Studies with chimeras of the gonadotropin receptors reveal the importance of third intracellular loop threonines on the formation of the receptor/nonvisual arrestin complex. Biochemistry. 2003;42:13950–139509. 14636063 10.1021/bi034907w

[79] Menon KMJ, Clouser CL, Nair AK. Gonadotropin receptors: role of post-translational modifications and post-transcriptional regulation. Endocrine. 2005;26: 249–257. 16034179 10.1385/ENDO:26:3:249

[80] Munshi UM, Clouser CL, Peegel H, Menon KM. Evidence that palmitoylation of carboxyl terminus cysteine residues of the human luteinizing hormone receptor regulates postendocytic processing. Mol Endocrinol. 2005;19: 749–58. 15539429 10.1210/me.2004-0335

[81] Uribe A, Zariñán T, Pérez-Solis MA, Gutiérrez-Sagal R, Jardón-Valadez E, Piñeiro Á, et al Functional and Structural Roles of Conserved Cysteine Residues in the Carboxyl-Terminal Domain of the Follicle-Stimulating Hormone Receptor in Human Embryonic Kidney 293 Cells. Biol Reprod. 2008; 78: 869–882. 18199880 10.1095/biolreprod.107.063925

[82] Ulloa-Aguirre A, Crépieux P, Poupon A, Maurel MC, Reiter E. Novel pathways in gonadotropin receptor signaling and biased agonism. Rev Endocr Metab Disord. 2011;12: 259–274. 10.1007/s11154-011-9176-2 21526415

[83] Salmon C, Nunez-Rodriguez J, Marchelidon J, Le Menn F, Fontaine YA. Gonadotropin binding sites in eel ovary: Autoradiographic visualization and new data on specificity. Reprod Nutr Dévelop. 1988;28: 1165–1175. 10.1051/rnd:19880712

[84] Nyuji M, Kitano H, Shimizu A, Lee JM, Kusakabe T, Yamaguchi A, et al Matsuyama M. Characterization, localization, and stage-dependent gene expression of gonadotropin receptors in chub mackerel (*Scomber japonicus*) ovarian follicles. Biol Reprod. 2013;88: 148 10.1095/biolreprod.112.107292 23636810

[85] Kobayashi T, Pakarinen P, Torgersen J, Huhtaniemi I, Andersen Ø. The gonadotropin receptors FSH-R and LH-R of Atlantic halibut (*Hippoglossus hippoglossus*) -2. Differential follicle expression and asynchronous oogenesis. Gen Comp Endocrinol. 2008;156: 595–602. 10.1016/j.ygcen.2008.02.010 18377904

[86] Andersson E, Nijenhuis W, Male R, Swanson P, Bogerd J, Taranger GL, et al Pharmacological characterization, localization and quantification of expression of gonadotropin receptors in Atlantic salmon (*Salmo salar* L.) ovaries. Gen Comp Endocrinol. 2009;163: 329–339. 10.1016/j.ygcen.2009.05.001 19442667

[87] García-López A, Bogerd J, Granneman JC, van Dijk W, Trant JM, Taranger GL, et al Leydig cells express follicle-stimulating hormone receptors in African catfish. Endocrinology. 2009;150: 357–65. 10.1210/en.2008-0447 18755797 PMC2732288

[88] Chauvigné F, Verdura S, Mazón MJ, Duncan N, Zanuy S, Gómez A, et al Follicle-stimulating hormone and luteinizing hormone mediate the androgenic pathway in Leydig cells of an evolutionary advanced teleost. Biol Reprod. 2012;87: 35 10.1095/biolreprod.112.100784 22649073

[89] García-López A, de Jonge H, Nóbrega RH, de Waal PP, van Dijk W, Hemrika W, et al Studies in zebrafish reveal unusual cellular expression patterns of gonadotropin receptor messenger ribonucleic acids in the testis and unexpected functional differentiation of the gonadotropins. Endocrinology 2010;151: 2349–2360. 10.1210/en.2009-1227 20308533 PMC2869266

[90] Chauvigné F, Zapater C, Gasol JM, Cerdà J. Germ-line activation of the luteinizing hormone receptor directly drives spermiogenesis in a nonmammalian vertebrate. Proc Natl Acad Sci U S A. 2014;111: 1427–32. 10.1073/pnas.1317838111 24474769 PMC3910593

[91] Rocha A, Gomez A, Zanuy S, Cerda-Reverter JM, Carrillo M. Molecular characterization of two sea bass gonadotropin receptors: cDNA cloning, expression analysis, and functional activity. Mol Cell Endocrinol. 2007;272: 63–76. 10.1016/j.mce.2007.04.007 17543442

[92] Mu WJ, Wen HS, He F, Li JF, Liu M, Zhang YQ, et al Cloning and expression analysis of follicle-stimulating hormone and luteinizing hormone receptor during the reproductive cycle in Korean rockfish (*Sebastes schlegeli*). Fish Physiol Biochem. 2013;39: 287–298. 10.1007/s10695-012-9699-9 22843313

[93] Lei ZM1, Rao CV, Kornyei JL, Licht P, Hiatt ES. Novel expression of human chorionic gonadotropin/luteinizing hormone receptor gene in brain. Endocrinology. 1993;132: 2262–2270. 8477671 10.1210/endo.132.5.8477671

[94] You S1, Kim H, Hsu CC, El Halawani ME, Foster DN. Three different turkey luteinizing hormone receptor (tLH-R) isoforms I: characterization of alternatively spliced tLH-R isoforms and their regulated expression in diverse tissues. Biol Reprod. 2000;62: 108–116. 10611074 10.1095/biolreprod62.1.108

[95] Yang E-J, Nasipak BT, Kelley DB. Direct action of gonadotropin in brain integrates behavioral and reproductive functions. Proc Natl Acad Sci USA 2007;104: 2477–2482. 17284605 10.1073/pnas.0608391104 PMC1893001

[96] Hu L, Wada K, Mores N, Krsmanovic LZ, Catt KJ. Essential role of G protein-gated inwardly rectifying potassium channels in gonadotropin-induced regulation of GnRH neuronal firing and pulsatile neurosecretion. J Biol Chem. 2006;281: 25231–25240. 16825187 10.1074/jbc.M603768200

[97] AL-Hader AA, Lei ZM, Rao CV. Neurons from fetal rat brains contain functional luteinizing hormone/chorionic gonadotropin receptors. Biol Reprod. 1997;56: 1071–1076. 9160703 10.1095/biolreprod56.5.1071

[98] Ohkubo M, Yabu T, Yamashita M, Shimizu A. Molecular cloning of two gonadotropin receptors in mummichog *Fundulus heteroclitus* and their gene expression during follicular development and maturation. Gen Comp Endocrinol. 2013;184: 75–86. 10.1016/j.ygcen.2012.12.019 23337032

[99] Dukic-Stefanovic S, Walther J, Wosch S, Zimmermann G, Wiedemann P, Alexander H, et al Chorionic gonadotropin and its receptor are both expressed in Human retina, possible implications in normal and pathological conditions. PloS One 2012;7: e52567 10.1371/journal.pone.0052567 23285091 PMC3526580

[100] Cui H1, Zhao G, Liu R, Zheng M, Chen J, Wen J. FSH stimulates lipid biosynthesis in chicken adipose tissue by upregulating the expression of its receptor FSHR. J Lipid Res. 2012;53: 909–917. 10.1194/jlr.M025403 22345708 PMC3329390

[101] Kobayashi Y, Nakamura M, Sunobe T, Usami T, Kobayashi T, Manabe H, et al Sex change in the Gobiid fish is mediated through rapid switching of gonadotropin receptors from ovarian to testicular portion or vice versa. Endocrinology. 2009;150: 1503–1511. 10.1210/en.2008-0569 18948407

