# Supplementary figures and images for: Demonstration of the Coexistence of Duplicated LH Receptors in Teleosts, and Their Origin in Ancestral Actinopterygians
Source: PLoS One. 2015 Aug 13;10(8):e0135184. doi: 10.1371/journal.pone.0135184 (PMC4536197; doi:10.1371/journal.pone.0135184)

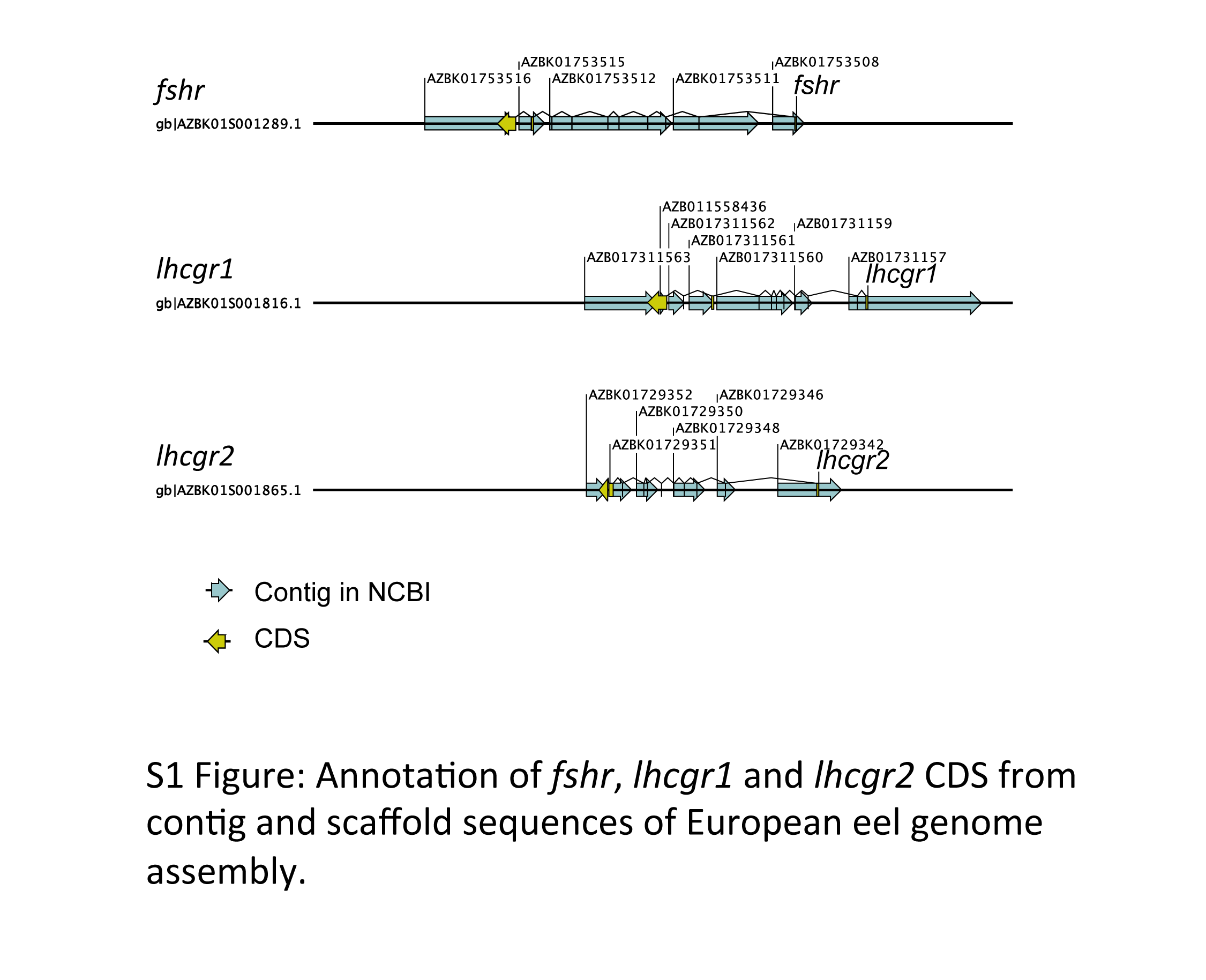

Supplement: S1 Fig — (TIF) [file pone.0135184.s001.tif]

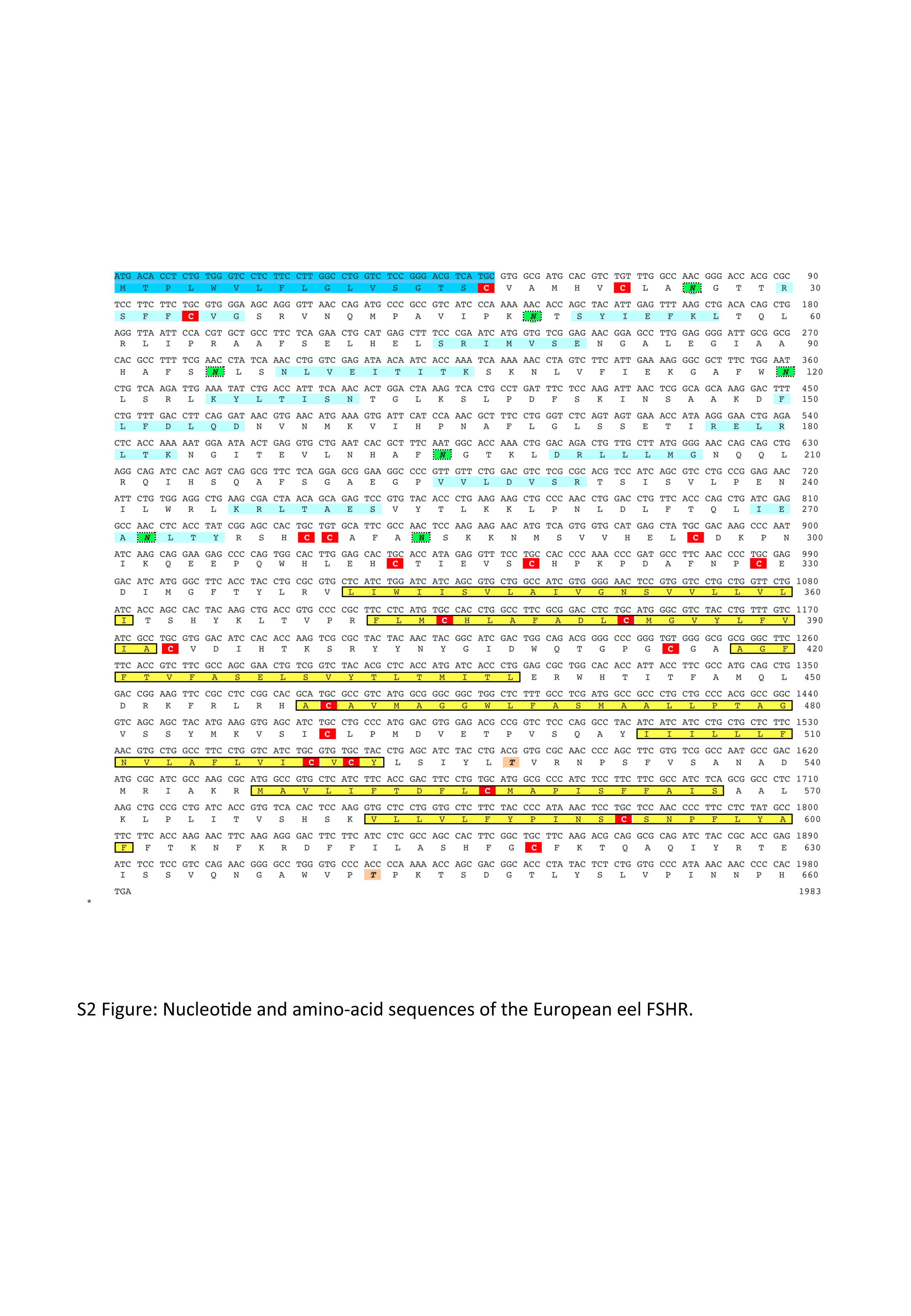

Supplement: S2 Fig — Nucleotide and deduced amino acid sequence of the eel fshr CDS. Numbers on the left refer to position of the nucleotide residues (top) and the amino acid (bottom). The predicted signal peptide is indicated in bold italics. Cysteine residues are indicated by red boxes. Putative sites for N-linked glycosylation are indicated by grey boxes. The eleven β-strand motifs of the LRR, identified by Pfam Blast and sequence alignment with the human FSHR, are indicated in blue light boxes. The position of the seven predicted alpha-helices is shown as yellow boxes. Potential sites for PKC phosphorylation are indicated by orange boxes. (TIF) [file pone.0135184.s002.tif]

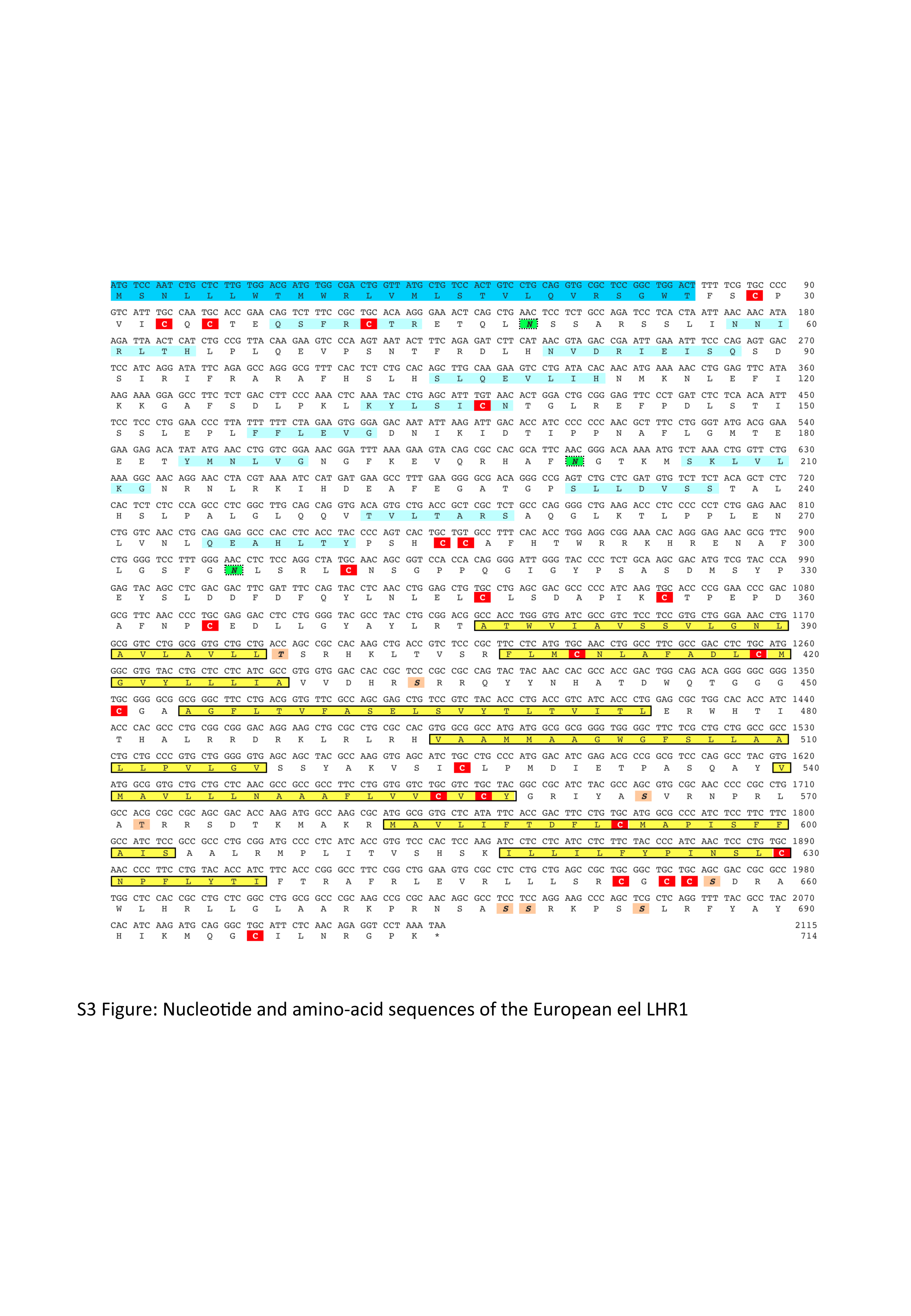

Supplement: S3 Fig — Nucleotide and deduced amino acid sequence of the eel lhcgr1 CDS. For symbols see legend of S3 Fig. (TIF) [file pone.0135184.s003.tif]

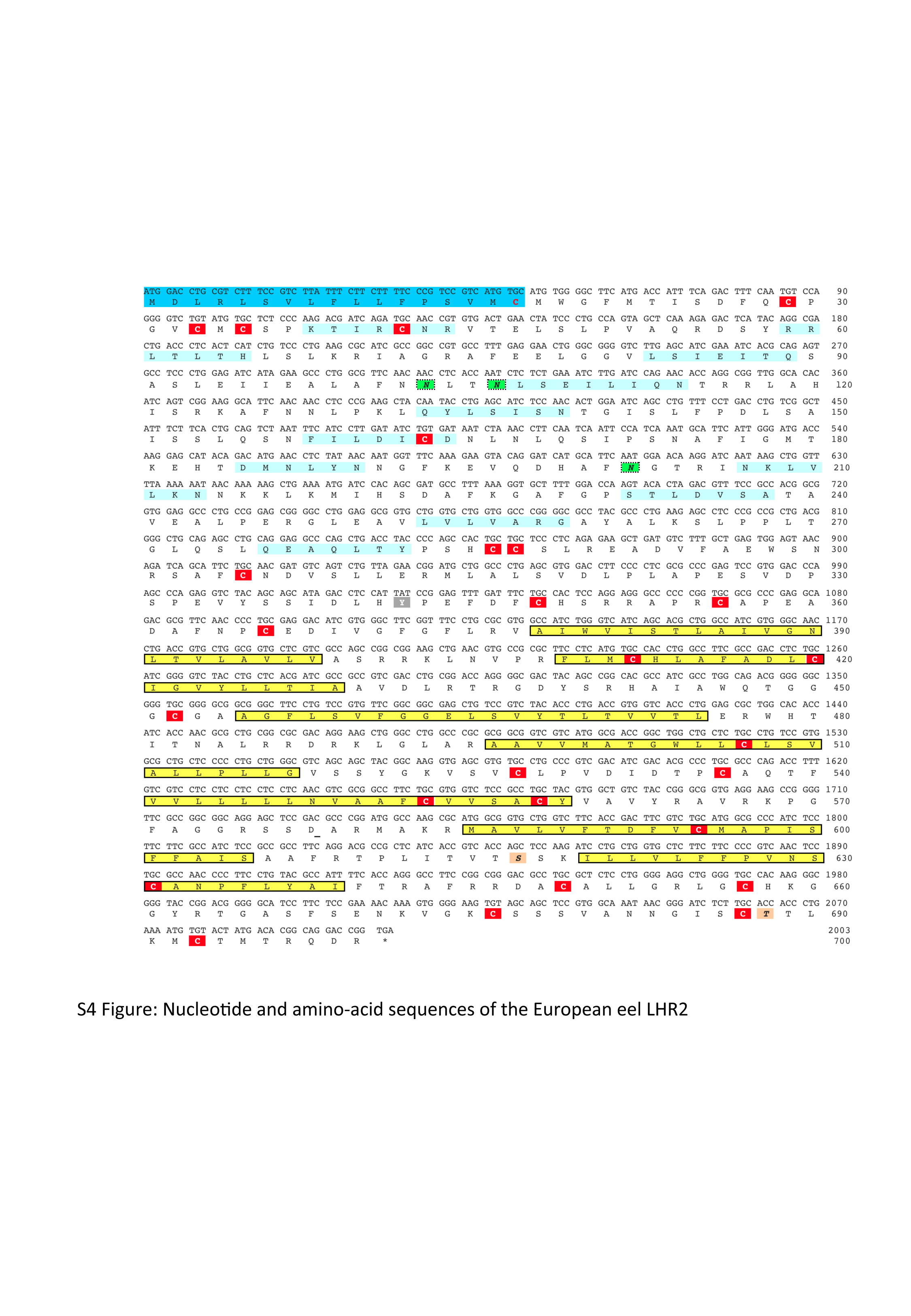

Supplement: S4 Fig — Nucleotide and deduced amino acid sequence of the eel lhcgr2 CDS. For symbols see legend of S3 Fig. (TIF) [file pone.0135184.s004.tif]

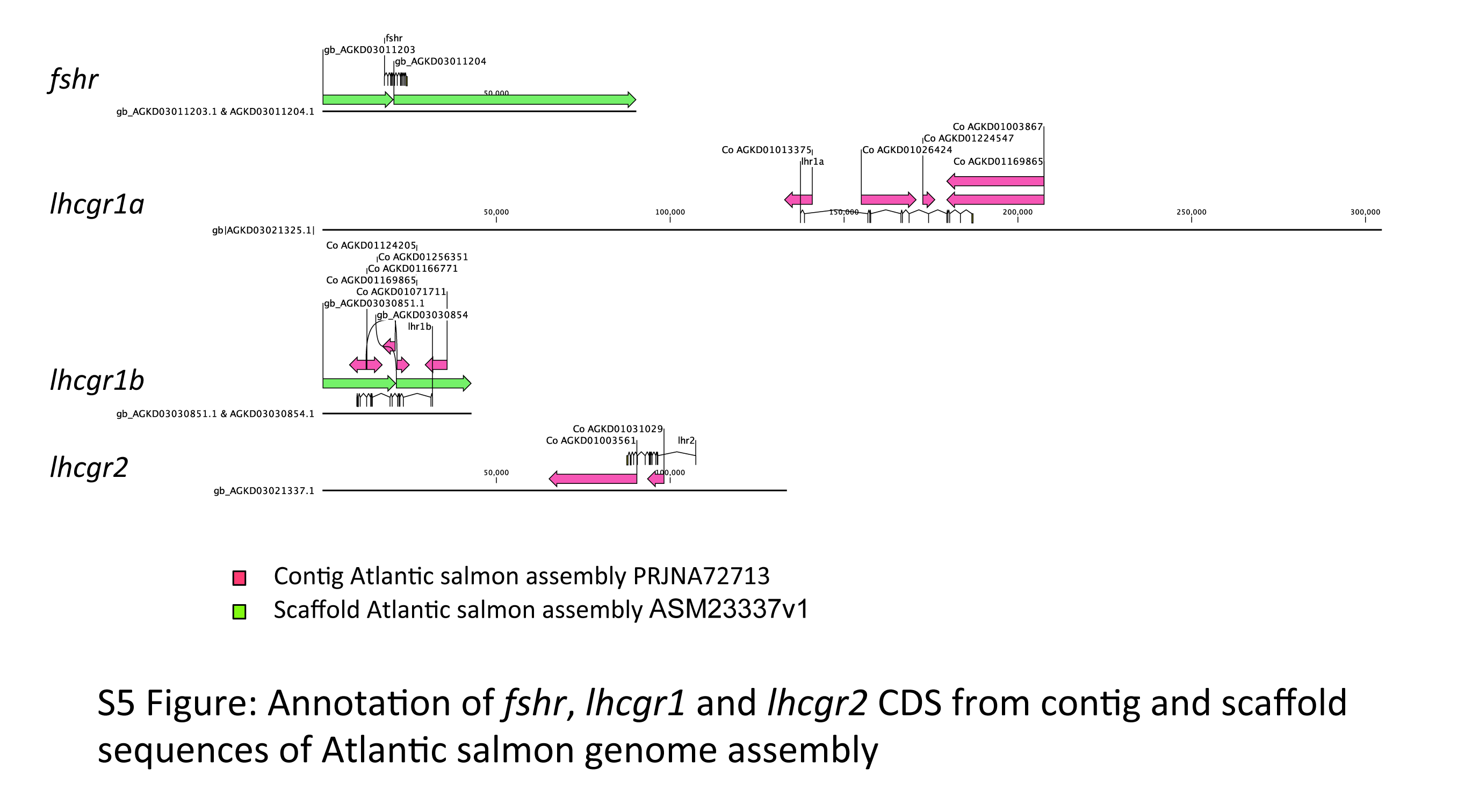

Supplement: S5 Fig — (TIF) [file pone.0135184.s005.tif]

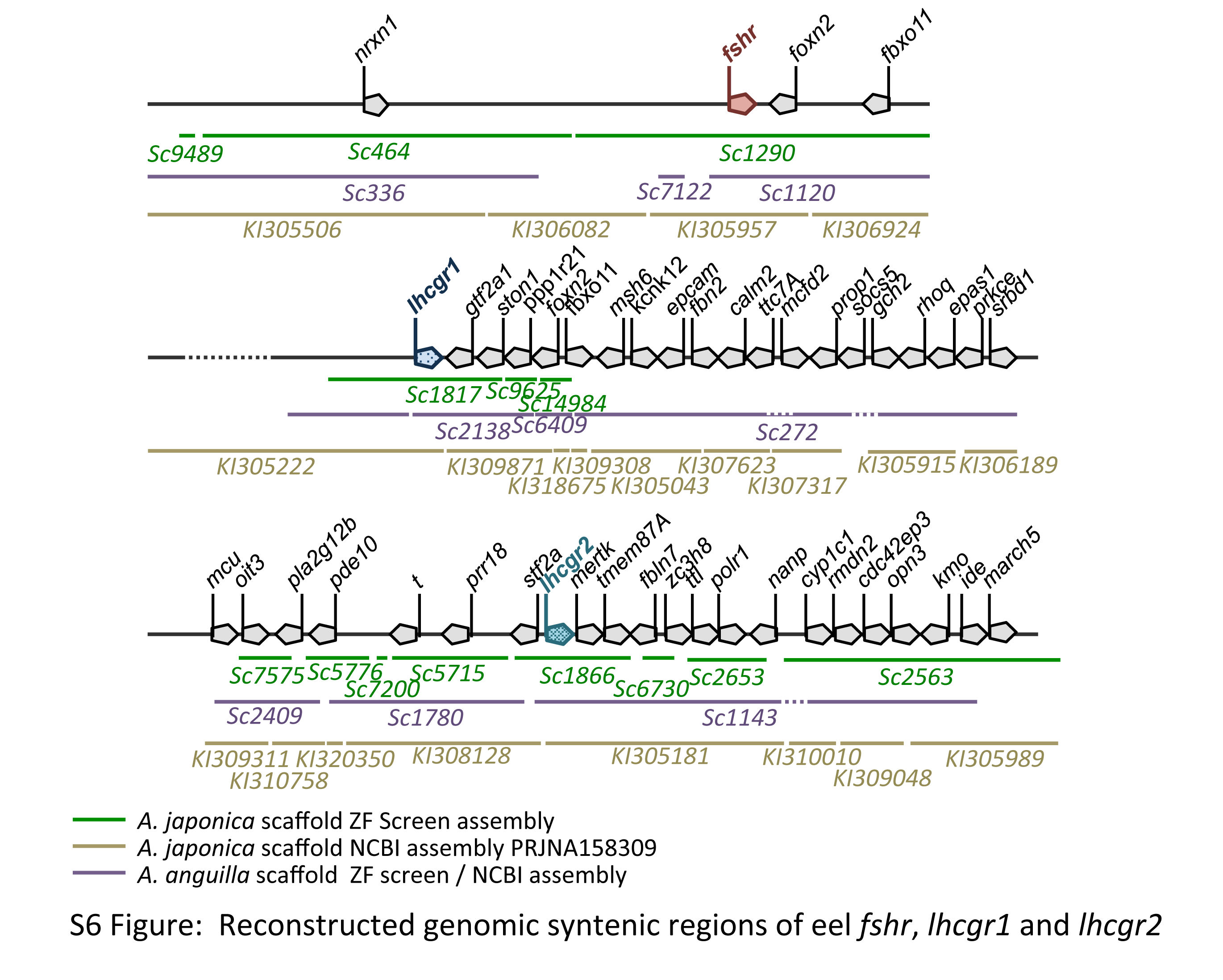

Supplement: S6 Fig — (TIF) [file pone.0135184.s006.tif]
