# Supplementary material for: Demonstration of the Coexistence of Duplicated LH Receptors in Teleosts, and Their Origin in Ancestral Actinopterygians
Source: PLoS One. 2015 Aug 13;10(8):e0135184. doi: 10.1371/journal.pone.0135184 (PMC4536197; doi:10.1371/journal.pone.0135184)
Supplement: S2 Table — (DOCX) [file pone.0135184.s009.docx]

S2 Table: Primer sets for cloning and quantitative real-time PCR of European eel gonadotropin receptors.

| Primers | Nucleotide sequences |
| --- | --- |
| Primers for cloning | |
| FSHRFW1 | 5’-CCT GAA GAA GCT GCC CAA CC -3’ |
| FSHRFW2 | 5’-GGT CGA GAT AAC AAT CAC CAA A -3’ |
| FSHRFW3 | 5’-CTC TTC CTT GGC CTG GTC TC -3’ |
| FSHRFW5 | 5’-ACC CCC ACA CTT GGA TCT AT-3’ |
| FSHRRV1 | 5’-GCC ATR CAC AGG AAG TCG GTG A-3’ |
| FSHRRV2 | 5’-AGG CGA TGA CAA ACA GGT AGA-3’ |
| FSHRRV3 | 5’-GGC TCC GAT AGG TGA GGT TG-3’ |
| FSHRRV5 | 5’-CCA TGG TTT TAC TGA AGG CTG T-3’ |
|  |  |
| LHR1FW1 | 5’-CAG GGA GAA CGC GTT CCT GG-3’ |
| LHR1FW2 | 5’-GAA AGG CAA CAG GAA CCT ACG-3’ |
| LHR1FW3 | 5’-GCC GTT ACA AGA AGT CCC AAG-3’ |
| LHR1FW3 | 5’-TGC CAA TGC ACC GAA CAG-3’ |
| LHR1RV1 | 5’-CGT AGC AGA CGC AGA CCAC-3’ |
| LHR1RV2 | 5’-GAC ACC AGG ATG GCC AAG-3’ |
| LHR1RV3 | 5’-GCG ATG AGG AGC AGG TAC AC-3’ |
| LHR1RV4 | 5’-GAC ACG GAA AGT GTG GAC CTC-3’ |
| LHR1RV5 | 5’-AGG CGT AAA ACC TGA GCG-3’ |
|  |  |
| LHR2FW1 | 5’-ATG GAC CTG CGT CTT TCC GTC T-3’ |
| LHR2FW2 | 5’-TCT TTT CCC GTC CGT CAT GTG C-3’ |
| LHR2FW3 | 5’-GAT GCA ACC GTG TGA CTG AA-3’ |
| LHR2FW4 | 5’-CCA TCT GGG TCA TCA GCA C-3’ |
| LHR2FW5 | 5’-ACG CCC TGA AGA GCC TCC CG-3’ |
| LHR2RV1 | 5’-CTG TAG ACC TCT GGG CTT GG-3’ |
| LHR2RV2 | 5’-AAC ACG GAC AGG AAG CCC GC-3’ |
| LHR2RV3 | 5’-CCG GCT GGC GAC GAG CAC C-3’ |
| LHR2RV4 | 5’-ATT GCA GCT TCG GGA GGT TGT-3’ |
| LHR2RV5 | 5’-CAG AGA GGA CGA TCC AAT CAC C-3’ |
|  |  |
| Primers for quantitative PCR | |
| RTFSHRFW | 5’-CCT GGT CGA GAT AAC AAT CAC C-3’ |
| RTFSHRRV | 5’-AAT CTT GGA GAA ATC AGG CAG T-3’ |
| RTLHR2FW | 5’-GCG GAA ACA CAG GGA GAA C-3’ |
| RTLHR2RV | 5’-GGT TGA GGT ACT GGA AAT CGA AG-3’ |
| RTLHR1FW | 5’-GTT TCC TGA CCT ATC GGC TAT T-3’ |
| RTLHR1RV | 5’-AGG TTC ATG TCT GTA TGC TCC TT-3’ |
| RTACTNFW | 5’-CAG CCT TCC TTC CTG GGT-3’ |
| RTACTNRV | 5’-AGT ATT TGC GCT CGG GTG-3’ |
